# Discerning Apical and Basolateral Properties of HT-29/B6 and IPEC-J2 Cell Layers by Impedance Spectroscopy, Mathematical Modeling and Machine Learning

**DOI:** 10.1371/journal.pone.0062913

**Published:** 2013-07-01

**Authors:** Thomas Schmid, Martin Bogdan, Dorothee Günzel

**Affiliations:** 1 Department of Mathematics and Computer Science, Universität Leipzig, Leipzig, Germany; 2 Institute of Clinical Physiology, Charité Universitätsmedizin Berlin, Berlin, Germany; University of Chicago, United States of America

## Abstract

Quantifying changes in partial resistances of epithelial barriers *in vitro* is a challenging and time-consuming task in physiology and pathophysiology. Here, we demonstrate that electrical properties of epithelial barriers can be estimated reliably by combining impedance spectroscopy measurements, mathematical modeling and machine learning algorithms. Conventional impedance spectroscopy is often used to estimate epithelial capacitance as well as epithelial and subepithelial resistance. Based on this, the more refined two-path impedance spectroscopy makes it possible to further distinguish transcellular and paracellular resistances. In a next step, transcellular properties may be further divided into their apical and basolateral components. The accuracy of these derived values, however, strongly depends on the accuracy of the initial estimates. To obtain adequate accuracy in estimating subepithelial and epithelial resistance, artificial neural networks were trained to estimate these parameters from model impedance spectra. Spectra that reflect behavior of either HT-29/B6 or IPEC-J2 cells as well as the data scatter intrinsic to the used experimental setup were created computationally. To prove the proposed approach, reliability of the estimations was assessed with both modeled and measured impedance spectra. Transcellular and paracellular resistances obtained by such neural network-enhanced two-path impedance spectroscopy are shown to be sufficiently reliable to derive the underlying apical and basolateral resistances and capacitances. As an exemplary perturbation of pathophysiological importance, the effect of forskolin on the apical resistance of HT-29/B6 cells was quantified.

## Introduction

Transepithelial resistence (R^T^, often also abbreviated as TER) is a standard parameter determined in epithelial physiology to characterize the properties of a given epithelium. R^T^ is usually determined under direct current (DC) or “near DC” (low frequencies to avoid electrode polarization) conditions [1]. Many components contribute to the R^T^ value of an epithelium: the resistance of subepithelial tissues, apical and basolateral membranes of the epithelial cells, and the paracellular cleft together with its sealing structure, the tight junction. In contrast to R^T^, the epithelial impedance Z^T^ is the opposition to alternating currents (AC). Generally, impedances of electric circuits are frequency-dependent, if they contain electrical components (e.g. capacitors, inductors) that cause phase shifts between current and voltage, and impedance spectroscopy analyzes this frequency dependence.

**Figure 1:**
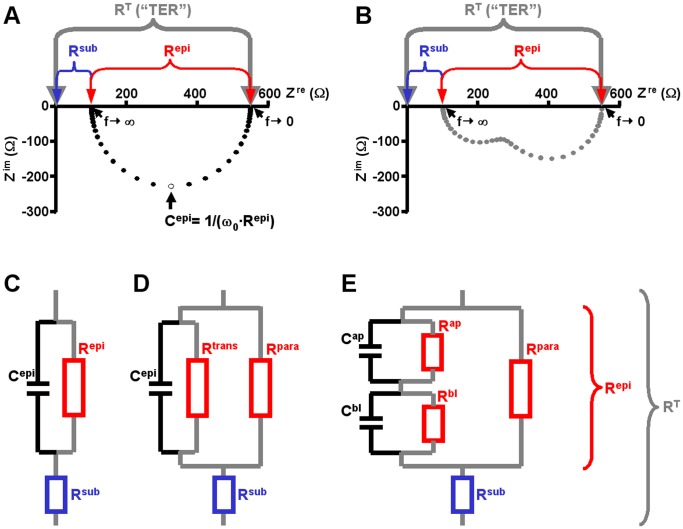
Nyquist diagrams and equivalent electrical circuits. **A)** Nyquist diagram of an impedance spectrum calculated for circuits depicted in (C) and (D). Data points, •, were calculated using 42 different frequencies between 1.3 Hz to 16 kHz. x-intercepts at low frequencies (f→0) correspond to the total epithelial resistance (R^T^, also called “TER”). x-intercepts at high frequencies (f→∞) correspond to the subepithelial resistance, R^sub^, as under these conditions the reactance of the membrane capacitor (1/(ω·C^epi^)) approaches zero and thus short-circuits R^epi^. Note that circuits (C) and (D) always yield semicircular spectra. **B)** Example for a Nyquist diagram of a non-semicircular impedance spectrum calculated for the circuit depicted in (E). Data points, **▴**, were calculated using 42 different frequencies between 1.3 Hz to 16 kHz. Spectra calculated for this model are the sum of two semicircles. Again, x-intercepts at low frequencies (f→0) correspond to the total epithelial resistance (R^T^), x-intercepts at high frequencies (f→∞) correspond to the subepithelial resistance, R^sub^. **C-E)** Equivalent electric circuits of epithelia. Components contributing to Repi are drawn in red, R^sub^ is highlighted in blue. Components contributing to R^T^ (sum of R^epi^ and R^sub^) are joint by grey lines. **C)** Simplest form of an equivalent electric circuit describing epithelial and subepithelial resistance (R^epi^, R^sub^) and epithelial capacitance (C^epi^). **D)** Equivalent electric circuit as in (C), but R^epi^ consists of two resistors in parallel, the transcellular (R^trans^) and the paracellular resistance (R^para^). **E)** Equivalent electric circuit as in (D), but the transcellular pathway is devided into an apical and a basolateral RC unit (R^ap^, C^ap^ and R^bl^, C^bl^, respectively).

In physiology, impedance spectroscopy has been employed for more than a century to investigate properties of plasma membranes and of epithelia [2, 3; for review see e.g. 1], utilizing the fact that biological membranes electrically act as resistor–capacitor circuits (RC circuits). Frequency response analyzers are employed to detect the phase angle θ between transepithelial current and voltage as well as the impedance magnitude |Z|. θ and |Z| may be plotted as polar coordinates, however, for convenience, these coordinates are usually converted into cartesian coordinates by employing complex numbers (see Supporting Information, Fig. S1; File S1, Eqs. S1– S5). Consequently, impedance spectra (typically using AC frequencies, f, between ∼1 Hz and ∼100 kHz) are often presented as Nyquist diagrams (Fig. 1A and 1B), in which the real (Z^re^) and the imaginary part (Z^im^) of the complex impedance are plotted against each other. Negative Z^im^ values reflect the fact that capacitors cause a phase shift of −90°, i.e. that the voltage lags the current (for a more detailed derivation see e.g. [1]).

Epithelia have been described by various equivalent electric circuits of different degrees of complexity. In one of the simplest equivalent circuits (Fig. 1C), three components are used: the epithelial resistance (R^epi^) and the epithelial capacitance (C^epi^) in parallel, and the subepithelial resistance (R^sub^) in series to these two elements. In a Nyquist diagram, this model yields semicircular impedance spectra and all three parameters can be derived directly from this diagram: R^sub^ as the intercept of the spectra with the x-axis at highest frequencies, R^epi^ as the diameter of the semicircle and C^epi^ from the angular frequency ω_0_ ( = 2·π·f_0_) at which Z^im^ reaches its minimum (C^epi^ = 1/(ω_0_·R^epi^); [4]; Fig. 1A).

A four-parameter model further divides R^epi^ into two parallel components, the paracellular (R^para^) and transcellular (R^trans^) resistance (Fig. 1D). These resistances cannot be derived directly from Nyquist diagrams. They can be determined, however, if one of them is experimentally altered by a known amount [5]. In an approach dubbed “two-path impedance spectroscopy” (2PI), Krug et al. [5] modified R^para^ by reducing the free extracellular Ca^2+^ concentration ([Ca^2+^]_o_) through chelation with ethylene glycol tetraacetic acid (EGTA) and at the same time determined flux changes of the paracellular marker substance fluorescein. The technique has been successfully employed to quantify paracellular effects of alterations in the molecular composition of tight junctions [5]–[8] or of impairment of tight junction integrity [9]. The accuracy of this method, however, critically depends on the accurate determination of R^epi^ and thus of the two intercepts of the impedance spectra with the x-axis. As shown in File S2 (Eqs. S6–S13), deviations from the actual values of R^sub^ and R^epi^ do not simply add up, but induce a multiplicative error in the calculation of R^trans^ and R^para^.

Whereas for many cultured epithelial monolayers impedance spectra are near-semicircular, impedance spectra from native epithelia often greatly deviate from the semicircular shape [10]–[12]. Similar deviations are observed, if transcellular transport is activated in cultured epithelia and thus differences in the time constants, τ (τ = R·C), of the apical and basolateral membranes are induced [5], [12]. An even more detailed six-parameter model therefore distinguishes between electrical properties of the apical and basolateral membrane. In this model, R^trans^ is the sum of the apical (R^ap^) and basolateral resistance (R^bl^), C^epi^ consists of an apical (C^ap^) and basolateral (C^bl^) component with C^epi^ *= *C^ap^·C^bl^/*(*C^ap^+C^bl^) (“lumped model”; [5], [13]; see Fig. 1E). However, direct determination of all six circuit parameters is technically complex and involves e.g. intracellular recordings with glass microelectrodes [13]–[15]. In Nyquist diagrams, this model only yields semicircular impedance spectra, if the time constants of the two membranes, τ^ap^ and τ^bl^, are equal [16] (cf. Fig. 1A and 1B for τ^ap^ ≈ τ^bl^ and τ^ap^ ≠ τ^bl^, respectively).

In principle, 2PI can still be employed under such conditions [5], [17]. However, as evaluated here, conventional fits are inadequate to accurately determine x-intercepts of non-semicircular Nyquist diagrams, thus limiting 2PI applicability. To overcome this limitation, a computational approach employing techniques from the field of machine learning was chosen in the present study. The given task is a multi-dimensional non-linear regression task with unknown regression function. Therefore, we use artificial neural networks (ANNs), which are able to act as universal approximators. ANNs are complex hierarchical networks mimicking the information processing capabilities of nervous systems and consisting of abstract computing units called neurons [18]. Such units use mathematical functions and can be implemented in software or hardware. As these networks can be trained to recognize data patterns, a variety of ANN methodologies have proven to be useful in biotechnological, biochemical and microbiological applications [19], e.g. in classification of biological macromolecules [20] or in detection of viral and phage proteins [21]. Here, multilayer perceptrons were trained by standard backpropagation learning [18] to estimate R^sub^ and R^epi^ from model impedance spectra calculated using the six-component circuit (Fig. 1E) and a mathematical model of the setup-specific data scatter. Further, their ability to estimate these values from actually measured impedance spectra was assessed.

Both conventional estimates of R^sub^ and R^epi^ and ANN estimates were used to calculate R^trans^ and R^para^ for HT-29/B6 and IPEC-J2 cells under conditions that lead to non-semicircular impedance spectra. Benchmarking these approaches with model impedance spectra demonstrates that the employed ANNs predict R^sub^ and R^epi^ more accurately than conventional methods and thus allow more reliable R^trans^ and R^para^ estimates. R^para^ estimates were subsequently used to estimate apical and basolateral membrane resistances and capacitances by a combination of curve and parameter fittings. Experiments employing the secretagogue forskolin demonstrate that the present method will be advantageous in drug studies on epithelia during which considerable alterations in membrane properties are elicited.

## Materials and Methods

### Cell Culture

Epithelial cell lines (human colonic carcinoma cells HT-29/B6, derived from HTB-38, ATCC, [22]; porcine jejunal cells IPEC-J2, ACC701, DSMZ, [23]) were cultured at 37°C and 5% CO_2_ in humidified air, using RPMI 1640 (PAA Laboratories GmbH, Pasching, Austria; HT-29/B6) and DMEM/Ham’s F12 (Sigma, Taufkirchen, Germany, IPEC-J2) supplemented with 10% (v/v) fetal bovine serum (Biochrom AG, Berlin, Germany), 100 U/ml penicillin, 100 µg/ml streptomycin (Gibco BRL, Karlsruhe, Germany). For experiments, cells were seeded onto filter supports (HT-29/B6, Millicell PCF, 3 µm pore size; IPEC-J2 Millicell HA, 0.45 µm pore size, Millipore, Schwalbach, Germany) and grown to confluence within 6 (HT-29/B6) and 10 days (IPEC-J2).

### Two-path Impedance Spectroscopy

Confluent cell layers on filter supports were mounted in conventional Ussing chambers (custom-made, medical-technical laboratories, Charité Berlin, Germany). Both hemi-chambers were filled with 10 ml standard bath solution (in mM: 140 Na^+^, 123.8 Cl^−^, 5.4 K^+^, 1.2 Ca^2+^, 1.2 Mg^2+^, 2.4 HPO_4_^2−^, 0.6 H_2_PO_4_^−^, 21 HCO_3_^−^, 10 D(+)-glucose, pH 7.4 when equilibrated with 5% CO_2_/95% O_2_ at 37°C). Transepithelial potentials were clamped to 0 mV. Impedance scans were obtained as previously described [4], [5], [24], using 5 µA/cm^2^ (IPEC-J2) or 25 µA/cm^2^ (HT-29/B6) effective sine-wave alternating current at 42 frequencies between 1.3 Hz and 16 kHz (see File S3). The resulting voltage changes were detected by a frequency response analyzer (PMS 1700, Newtons4th Ltd. together with a 1286 electrochemical interface, Solartron Schlumberger), yielding complex impedance values that were recorded on a personal computer. During experiments, R^trans^ was either left unaltered, modified by apical application of the adenylate cyclase activator forskolin (stock solution 10 mM in DMSO, final concentration 10 µM) or modified by apical or basolateral application of the ionophore nystatin (stock solution 3 mg/100 µl DMSO, final concentration range 15–60 µg/ml). Subsequently, R^para^ was altered by reducing [Ca^2+^]_o_ (addition of EGTA, final concentration 1.3 mM). Changes in R^para^ were monitored throughout the entire experiment by assessing the unidirectional fluorescein flux as described by [5].

### Conventional Approaches to Estimate R^sub^ and R^epi^

#### Method M1

R^sub^ was estimated as the minimum value of Z^re^ obtained at the highest available frequencies. R^epi^ was calculated as the difference between the maximum Z^re^ obtained at the lowest available frequencies and the estimated R^sub^. The rationale behind this approach is the fact that for a semicircular impedance spectrum, the error will be <1% if the time constant τ of the epithelium lies within a range of 10/ω_min_ and 1/(10·ω_max_), i.e. between about 0.1 and 10 ms for the frequency range used in the present study. Errors would thus be dominated by data scatter due to electric noise.

#### Method M2

Cole-Cole fits were carried out on data points in Nyquist diagrams as originally described by [3]. Data points were fitted with circular arcs by minimizing the sum of squared residuals [see 4, 25]. Deviation of spectra from the semicircular shape was compensated for by moving the midpoint of the arc above the x-axis. The intercept of the arc with the x-axis at the high frequency end of the plot was taken as R^sub^, the length of chord between the two intercepts as R^epi^.

### Error Model

Data scatter intrinsic to the electrophysiological set-up was determined by mounting artificial membranes (polypropylene; thickness, 0.2 mm; area, 0.6 cm^2^; capacitance in the order of 10 pF/cm^2^) in Ussing chambers. Membranes were pierced to obtain DC resistances between 30 and 25,000 Ω cm^2^. 30–40 consecutive impedance spectra were recorded for each resistance (see Fig. S2). Standard deviations (SDs) of the resulting Z^re^ and Z^im^ were calculated for each frequency and expressed as % of the DC resistance value.

To model impedance spectra, data scatter was generated separately for real and imaginary parts as function of corresponding frequency and transepithelial resistance R^T^ and added to the computationally generated spectra [26]. Frequency dependence was approximated by non-linear regression of SDs at the 42 frequencies employed during physiological experiments and at a fixed R^T^ (≈500 Ω·cm^2^). For Z^re^, a second-order Fourier series was assumed as best fit, for Z^im^, a fourth-order polynomial function (for details see File S4, Eqs. S14–S15). To account for the dependence of data scatter on R^T^, dynamics of the SDs at 1.3 Hz were approximated by exponential functions (separately for real and imaginary parts, for details see File S4, Eqs. S16–S17). Data scatter induced by the capacitance of the membrane was assumed to be negligible.

### Generation of Neural Network Training Data

A total of eight datasets was created. To estimate R^sub^ of HT-29/B6 cell layers, two training datasets were created that were optimized for high (>250 Ω·cm^2^) or low (<250 Ω·cm^2^) R^epi^ values, reflecting conditions before and after EGTA-induced Ca^2+^ switch. Using these datasets, two ANNs were trained to estimate R^sub^ for HT-29/B6 cells before (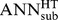) and after EGTA-induced Ca^2+^ switch (
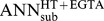
). Similarly, for determination of R^epi^ for HT-29/B6 cell layers before and after Ca^2+^ switch, two further training datasets and ANNs were employed (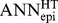,
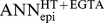
). Analogously, four datasets and ANNs were generated for IPEC-J2 cell layers (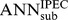
and 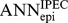 for R^epi^ >600 Ω·cm^2^; 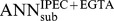 and 
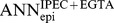
 datasets for R^epi^ <600 Ω·cm^2^). For an overview of all datasets created and used for ANN training see Supporting Information (File S3; Tables S1, S2, S3, S4).

#### Calculations

Based on the six-component circuit (Fig. 1E) and the AC frequencies applied during experiments, model impedance spectra were calculated. For each impedance spectrum, n = 42 frequency-dependent impedances Z^T^ were calculated for frequencies ω_1_ to ω_n_ (cf. File S3), establishing the following relation:
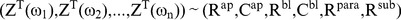
(1)where the complex number Z^T^ can be expressed for the assumed electric circuit as



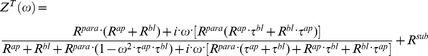
(2)τ^ap^ = R^ap^ · C^ap^ and τ^bl^ = R^bl^ · C^bl^ are introduced for simplicity; 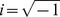. Real and imaginary parts Z^re^(ω) and Z^im^(ω) were calculated according to Eqs. S4 and S5 (cf. File S1). To each impedance value, i.e. to each real and imaginary part, a random value was added which matched the error model for the electrophysiological setup (cf. File S4, Eqs. S14–S17).

#### Sampling

To create training data for estimating R^epi^, values of all six underlying circuit components were reversely derived from a target value (chosen from a parameter range based on published values, cf. Table 1) in a consecutive manner for each model impedance spectrum. Starting with R^epi^, either R^para^ or R^trans^ were chosen to be greater than R^epi^ and within the absolute ranges for the circuit parameters (Table 1). For the remaining other parameter, the range was adjusted dynamically and a subset of ten equally distributed values was chosen. A similar procedure was applied to R^ap^ and R^bl^ (where R^ap^+R^bl^ = R^trans^). For capacitances C^ap^ and C^bl^, reasonable static ranges were estimated from published C^epi^ values (Table 1). To create training data for estimating R^sub^, training data produced for estimating R^epi^ were shifted by a random value matching the given ranges for R^sub^ (Table 1).

**Table 1:** Parameter ranges for HT-29/B6 and IPEC-J2 cell-appropriate training data.

	R^sub^ [Ω·cm^2^]	R^epi^ [Ω·cm^2^]	R^para^ [Ω·cm^2^]	R^trans^ = R^ap^+R^bl^ [Ω·cm^2^]	C^epi^ = 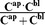 [Ω·cm^2^]	C^ap^ [µµF/cm^2^]	C^bl^ [µµF/cm^2^]
**HT-29/B6**							
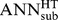	1–30 (Δ 0.5)	*250–1,250*	250–6,500	1–3,500	*2.5–7.5*	1–75	1–75
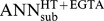	1–30 (Δ 0.5)	*1–250*	1–650	1–3,500	*2.5–7.5*	1–75	1–75
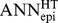	0	250–1,250 (Δ 3)	250–6,500	1–3,500	*2.5–7.5*	1–75	1–75
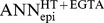	0	1–250 (Δ 1.5)	1–650	1–3,500	*2.5–7.5*	1–75	1–75
**IPEC-J2**							
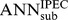	1–30 (Δ 0.5)	*600–10,000*	1–15,000	5,000–25,000	*1–5*	1–10	1–10
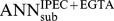	1–30 (Δ 0.5)	*1*–*600*	1–650	5,000–25,000	*1–5*	1–10	1–10
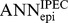	0	600–10,000 (Δ 25)	1–15,000	5,000–25,000	*1–5*	1–10	1–10
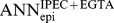	0	1–600 (Δ 2.5)	1–650	5,000–25,000	*1–5*	1–10	1–10

Δ values denote fixed step sizes used for the generation of model impedance spectra; in all other cases, step sizes were chosen dynamically in order to yield 10 steps per parameter. Values *in italics* denote absolute interval constraints rather than variables with varied values. Ranges for HT-29/B6 circuit parameters were based on published estimates: R^trans^ between 380 and 1500 Ω·cm^2^, R^para^ between 1500 and 4 000 Ω·cm^2^ which was reduced to between 20 and 100 Ω·cm^2^ at low [Ca^2+^]_o_, C^epi^ between 2.1 and 3.5 µF/cm^2^, R^sub^ (resistance of filter supports) between 2 and 10 Ω·cm^2^ [5], [24], [25]. IPEC-J2 cells possess very high R^epi^ (several k Ω·cm^2^, [47]). Literature values for any further parameter values were not available.

#### Cross-Validation

For each of the eight conditions, 50,000 of all spectra generated were selected randomly and split into a training dataset of 25,000 samples (50%) and a test dataset of 25,000 samples (50%). To allow monitoring progress during the training phase, the test dataset was used as validation dataset; these samples were neither used for weight adjustment nor as a stopping criterion during the training phase. For cross-validation, training and test datasets were interchanged (with initial conditions of the networks set to random).

### Architecture and Training of the Employed Neural Networks

To estimate R^sub^ or R^epi^ respectively, standard feed-forward neural networks were employed with standard backpropagation as learning algorithm. The network architectures were based on a total of three layers of neural units: input layer, hidden layer, output layer (Fig. S3). The activation functions of the neural units were uniform within each layer: linear for input and output layers, sigmoidal for the hidden layer. Learning rates varied between 0.00001 and 0.000025. In all cases, training was performed for a total of 10,000 epochs (Fig. S4).

A total of eight networks were employed. All networks were designed as a 20-2-1 architecture, i.e. the input layer consisted of 20 units, each of which can be seen as features or channels representing consecutively ordered real and imaginary parts of 10 impedances. The hidden layer consisted of 2 units possessing weighted connections to all input units and to the output unit. The output unit represented the estimated value of R^sub^ or R^epi^, respectively. Input units corresponded to frequencies 2060.3 Hz –16.3 kHz for ANNs targeting R^sub^ and to frequencies 1.3 Hz –10.3 Hz for ANNs targeting R^epi^.

### Benchmarking against Conventional Approaches

ANN estimations for R^sub^ and R^epi^ were compared with results of the reference methods M1 and M2 to assess both precision of the estimations (with modeled spectra) and plausibility of the electric model and the setup-specfic error model (with measured spectra).

#### Modeled spectra

For each target parameter (R^sub^ or R^epi^) under each condition (before or after EGTA application) and for each cell type, a subset of the corresponding test dataset was used as benchmark data. These modeled impedance spectra were not used as training data for the ANN. To separate semicircular curves (observed before application of nystatin or forskolin) from non-semicircular curves (observed afterwards), samples were divided into groups with a τ ratio (larger membrane time constant divided by smaller membrane time constant) being less than five or greater than five, respectively. The border value of five was chosen from model calculations [16], [26]. From both groups, 1000 spectra were chosen randomly and evaluated by all three methods (1: reference method M1; 2: reference method M2; 3: the corresponding ANN matching cell line, target parameter and the EGTA condition of the sample set from which the spectrum originates). Results were compared to the corresponding target values from which the modeled spectra had been calculated.

#### Measured spectra

To assess the accuracy of estimating R^sub^ and R^epi^ from experimental data, 393 impedance spectra from 38 experiments on HT-29/B6 cell layers and 192 impedance spectra from 13 experiments on IPEC-J2 cell layers were evaluated with methods M1, M2, and the ANN matching cell line, target parameter and EGTA condition. During these experiments, R^trans^ and/or R^para^ had been manipulated by the application of forskolin, nystatin (R^trans^ modulators) and/or EGTA (R^para^ modulator). Of 281 HT-29/B6 spectra recorded before EGTA application, 223 and 58 spectra were obtained in the absence and presence of R^trans^ modulators, respectively. Similarly, 112 HT-29/B6 spectra belonging to the EGTA-treated group were further divided into 79 and 33 spectra (additional absence and presence of R^trans^ modulators, respectively). Corresponding numbers for impedance spectra from IPEC-J2 cells were 166 before EGTA application (59 without, 107 with R^trans^ modulation) and 26 after EGTA application (11 without, 15 with R^trans^ modulation; see Table S5 for an overview of all measured datasets).

**Figure 2:**
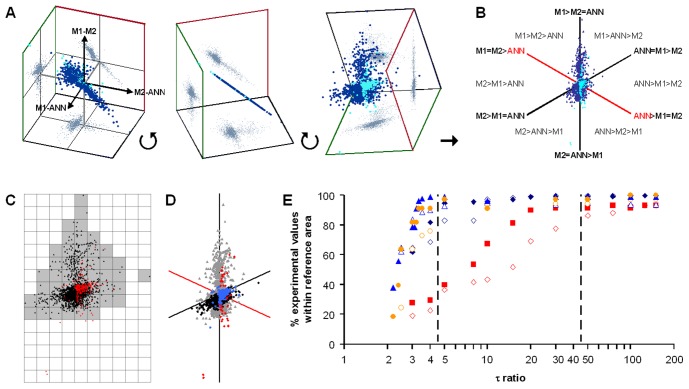
Similarity diagrams for the evaluation of M1, M2 and ANN precision. **A)** Differences between estimates (M1–M2, M1-ANN, M2-ANN) were calculated for experimental data (light blue) as well as for the 2×1000 benchmark spectra (dark blue) and normalized with respect to the estimated (measured spectra) or target (modeled spectra) R^epi^ values. Plotting these differences on the three axes of a three-dimensional coordinate system yielded a distribution with all data points falling onto a single plane within the three-dimensional space. Grey dots represent projections of the data points onto the three planes. 3D scatterplots were created with Excel macros provided by Gabor Doka (http://www.doka.ch/Excel3Dscatterplot.htm). **B)** Data points were transformed to present this plane in a two-dimensional similarity diagram with three axes. Data scatter along the red axis indicates a greater dissimilarity of values obtained with the appropriate ANN when method M1 and M2 yield very similar results. Data are derived from spectra generated for R^sub^ estimation of HT-29/B6 cells in the absence of EGTA. **C)** Shaded “reference” area, indicating the area covered by data points (black **▴**) shown in (A), was determined in a 10 by 10 grid. 276 of 281 (98.2%) of the data points from measured spectra (red ♦) are contained by the reference area, indicating that the model spectra were generated for appropriate parameter ranges (see also Fig. S7). **D)** Distribution of data points from modeled spectra with τ ratios <5 (black ♦) is similar to the distribution of data points from measured spectra (blue •) obtained in the absence of forskolin or nystatin. Conversely, distribution of data points from model spectra with τ ratios >5 (grey **▴**) is similar to the distribution of data points from measured spectra (red ♦) obtained in the presence of forskolin or nystatin. This indicates that τ ratios of measured spectra can be estimated from the distribution of data of model spectra with known τ ratios. Axes as in B. For all other conditions see Fig. S6. **E)** Estimating τ ratios of measured HT-29/B6 spectra before and during forskolin or nystatin application. Data points from spectra obtained in the absence of any drugs (no EGTA, nystatin or forskolin; blue ♦, R^epi^; blue **⋄**, R^sub^) showed highest similarity with those of modeled spectra with τ ratios ≤ 4.5. In contrast, τ ratios of up to ∼50 were needed to model data points from spectra obtained in the presence of forskolin or nystatin but absence of EGTA (red ▪, R^epi^; red □, R^sub^). If R^trans^ was short-circuited by an EGTA-induced decrease in R^para^, all spectra assumed near semicircular shapes, irrespective of the absence (blue **▴**, R^epi^+EGTA; blue **▵**, R^sub^+EGTA) or presence (yellow •, R^epi^+EGTA; yellow **○**, R^sub^+EGTA) of forskolin or nystatin.

Differences between estimates (Δ_M1,M2_, Δ_M1,ANN_, Δ_M2,ANN_) were calculated for experimental data as well as for the benchmark spectra and normalized with respect to the estimated (measured spectra) or target (modeled spectra) R^epi^ values. Plotting these differences on the three axes of a three-dimensional coordinate system yielded a distribution with all data points falling onto a single plane within the three-dimensional space (Fig. 2A). Data points were transformed to present this plane in a two-dimensional diagram with three axes (Fig. 2B).

The resulting diagrams (dubbed “similarity diagrams”) visualize similarity/dissimilarity of estimates obtained by the three methods. The diagrams have the following characteristics: (1) If estimates from all three methods are similar, data points lie close to the origin of the diagram. (2) If estimates from one method are more dissimilar from the estimates of the other two methods, data points will spread along one specific axis, as illustrated in Fig. 2B. It is assumed that ANNs have been trained for an appropriate range, if data points from measured spectra lie within the area covered by the data obtained from model spectra (reference area, Fig. 2C).

### Calculation of Apical and Basolateral Parameters

Two impedance spectra before and two after the application of nystatin or forskolin were chosen per experiment. The following assumptions were made: (1) Nystatin only affects the membrane resistance at the side of application (i.e. either R^ap^ or R^bl^). (2) Forskolin only affects R^ap^. (3) The membrane resistance on the side not affected by nystatin or forskolin (R^ap^ or R^bl^, as appropriate), R^sub^, C^ap^, and C^bl^ are identical for all four spectra.

For R^para^, estimates from 2PI evaluations were used. Z^re^(ω) and Z^im^(ω) were calculated for all 42 frequencies used during measurements (File S3; File S1, Eqs. S4 and S5) and a least*-*squares fitting approach was used to estimate R^sub^, R^ap^, R^bl^, C^ap^ and C^bl^ from measured impedance spectra. Model spectra were used to test the accuracy of this approach. Under these test conditions it was found that increasing deviations in R^para^ caused increases in the average deviation per impedance value, i.e., the best fit could only be obtained with the most accurate R^para^. The average deviation per impedance value between measured and fitted impedance values was therefore used to evaluate which of the three methods (M1, M2, ANN) yielded the most accurate values for R^sub^, R^ap^, R^bl^, C^ap^ and C^bl^.

### Data Evaluation/Statistics

R^sub^ and R^epi^ of 2000 benchmarking spectra calculated for each condition (HT, HT+EGTA, IPEC, IPEC+EGTA) were evaluated by all three methods (M1, M2, ANN). Differences between estimated R^epi^ and R^sub^ values and the corresponding target values from which the modeled spectra had been calculated were determined. These differences were found to be normally distributed when obtained with the appropriate ANN, however, values obtained with M2 and part of the values obtained with M1 (HT-epi, IPEC-epi, IPEC+EGTA-epi) were not normally distributed (Kolmogorov–Smirnov test, p<0.01). Therefore, the Wilcoxon signed-rank test was employed to determine, which method yielded the most accurate estimate.

For measured spectra, target values obviously are not available. Therefore, differences between values estimated by the three methods were calculated for measured as well as for modeled spectra and the distribution of these differences was compared in similarity diagrams, as outlined above.

All partial resistances and capacitances estimated for HT-29/B6 and IPEC cells are expressed as means±SEM and compared by using Student’s t-test (unpaired, two-tailed).

## Results

### Benchmarking against Conventional Methods

#### Modeled spectra

**Figure 3:**
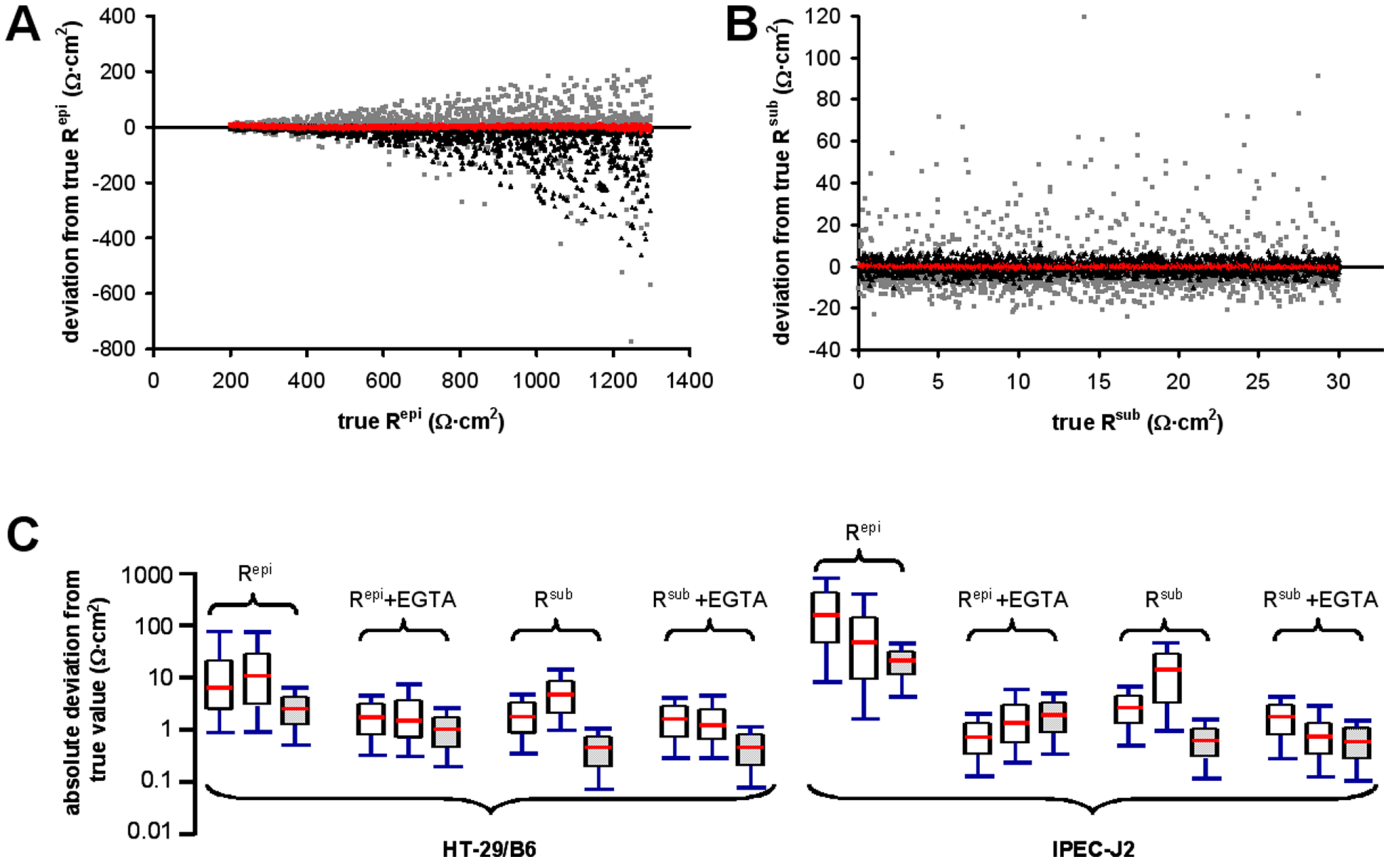
Comparison of M1, M2 and ANN precision with model impedance spectra. **A,B)** Deviations of estimated R^epi^ (A) and R^sub^ (B) values (estimated minus true value), using method M1 (grey ▪), M2 (black **▴**) and the appropriate ANN (red •) are plotted against the respective known target value. Values refer to HT-29/B6 cells in the absence of EGTA. For all other conditions see Fig. S5. **C)** Box plot of absolute deviations (|estimated minus true value|) for all eight conditions. Each data set was evaluated using M1 (left), M2 (middle) and the appropriate ANN (right, shaded). Midline: median; box, 1^st^ and 3^rd^ quartile; tails, minimum and maximum. Except for the 
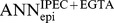
 which performs significantly worse than M1 (p<<0.01), all ANNs perform highly significantly better (p<<0.01) than M1 and M2 (Wilcoxon signed-rank test).

Fig. 3 A and B show the differences between target values and estimated values for 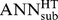 and 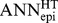 (see Fig. S5 for results under all other conditions). In all cases except one (
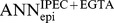
, best method: M1; Wilcoxon signed-rank test, p<<0.01), estimates from ANN were highly significantly better than for the two other methods (Wilcoxon signed-rank test, p<<0.01; see Fig. 3C).

#### Measured spectra

For the four ANNs generated for HT-29/B6 cells, 95% to 98% of all measured values were found to lie within the corresponding reference areas. Separate evaluation of the two subgroups of model impedance spectra with τ ratios ≤ 5 and >5, respectively, demonstrated that τ ratios correlated with the position of the data-points in the similarity diagrams, as values for lower τ ratios were found closer to the origin of the diagram than values for higher τ ratios (black vs. grey data points in Fig. 2D). In accordance with this observation, 95% of R^epi^ data and 83% of R^sub^ data from experiments without R^trans^ modulation and without EGTA application were contained by reference areas for τ ratios ≤ 5 (compare blue and black data points in Fig. 2D and Fig. S6). In contrast, only 40% and 36% of the respective data from experiments with R^trans^ modulation were contained by reference areas for τ ratios ≤ 5 (compare red and black data points in Fig. 2D and Fig. S6). Further analysis of this correlation at different τ ratios was used to estimate the increase of τ ratios under the experimental conditions used in the present study. As shown in Fig. 2E, τ ratios of cell layers increased by about a factor of 10 upon modulation of R^trans^.

No such correlation was observed for values from spectra obtained after EGTA treatment. These values reflect conditions, where the low R^para^ short-circuited R^trans^ and where, consequently, curves in Nyquist diagrams were semicircular despite alterations in R^ap^ or R^bl^ [16]. Consequently, 92% to 99% of all data points were contained by the reference areas for τ ratios ≤ 5 (see also Fig. 2E).

For data from IPEC-J2 cell layers, similar results were obtained, although agreement between modeled and measured data was generally weaker. For the four ANNs generated for IPEC-J2 cells, 80% to 100% of all estimates were found to lie within the reference area (all τ ratios). For data obtained before EGTA application, 75% (R^epi^) and 70% (R^sub^) of the estimates for spectra obtained without R^trans^ modulation were contained by the reference areas for τ ratios <5. Under R^trans^ modulation 70% (R^epi^) and 37% (R^sub^) lay within the reference areas.

Similar to results for HT-29/B6 cells, estimates for data obtained after the application of EGTA were contained by the reference areas for τ ratios <5, even if their R^trans^ had been altered during experiments (93% for both, R^epi^ and R^sub^). This was also true for R^sub^ values without R^trans^ manipulation (100%) but not for R^epi^ values (64%), possibly reflecting the lower precision of the
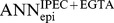
.

### Calculation of R^para^ and R^trans^

**Figure 4:**
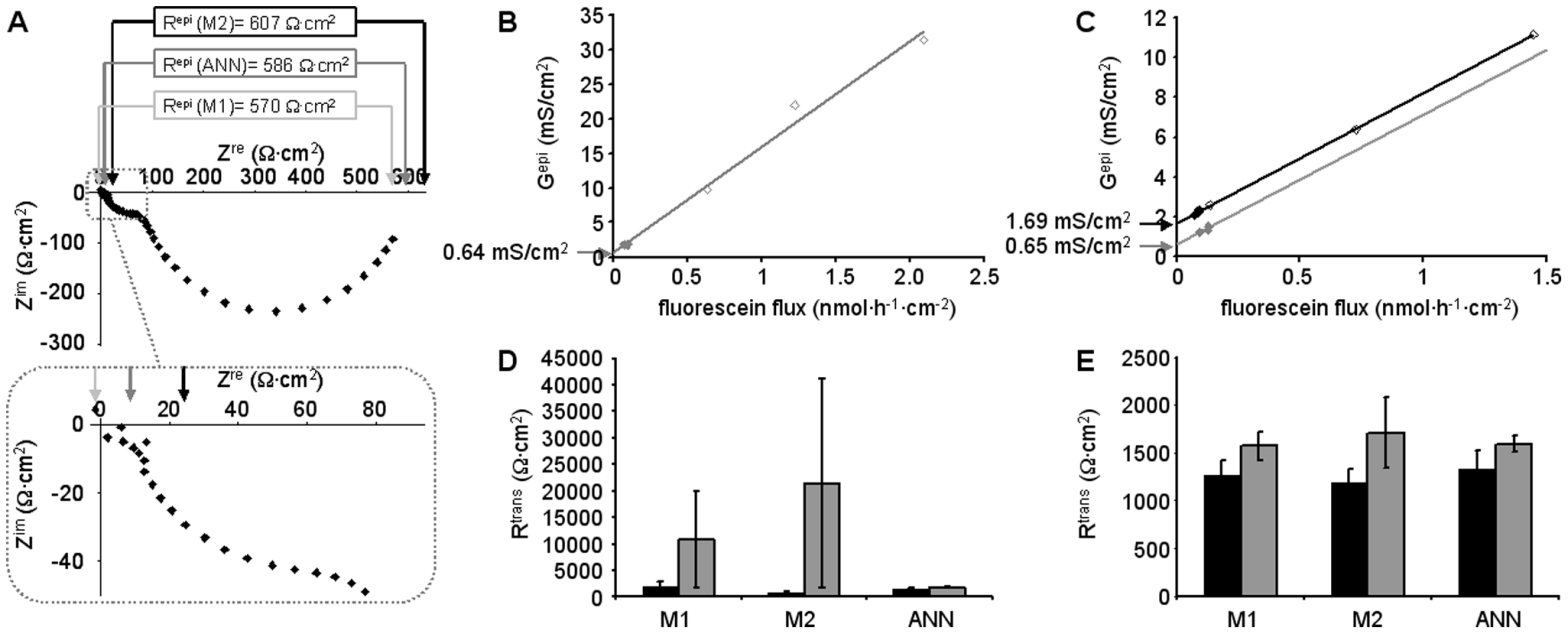
Applicability of 2PI under conditions of altered R^ap^ or R^bl^. **A)** Nyquist plot of HT-29/B6 impedance spectrum after the application of forskolin. Arrows indicate estimates of R^sub^ (see inset) and R^T^ using the three methods, M1 (light grey), M2 (dark grey) and ANN (black). R^T^ = R^epi^+R^sub^. **B,C)** 2PI: Plotting epithelial conductance G^epi^ = 1/R^epi^ (♦, in the absence; ♦, in the presence of forskolin; ⋄, after EGTA application) against transepithelial fluorescein flux allows estimate of transcellular conductance, G^trans^ [5]. (B) Experiment without forskolin application. G^trans^ equals y-intercept (arrow) of the linear regression (grey line, G^trans^ = 0.64 mS/cm^2^). (C) G^trans^ in the presence of forskolin is obtained from the y-intercept (black arrow) of the linear regression (black line, G^trans^ = 1.69 mS/cm^2^). Shifting the linear regression to pass through the values obtained before the application of forskolin (grey line) allows estimate of G^trans^ in the absence of forskolin (grey arrow, G^trans^ = 0.65 mS/cm^2^). **D)** Comparison of R^trans^ values obtained from 30 experiments (15 without and 15 with forskolin or nystatin application, black and grey bars, respectively), using the three methods to estimate R^epi^. Four experiments (three without and one with nystatin application) yielded negative or unreasonably high R^trans^ values when evaluated with methods M1 and M2. **E)** Same as (D) but after omission of these four experiments. Remaining estimates from experiments without forskolin or nystatin application were very similar for all three methods, estimates from experiments with forskolin or nystatin application showed lowest variance when evaluated by ANN.

2PI evaluation to estimate R^para^ and R^trans^ requires accurate determination of R^epi^. However, accuracy may be limited by data scatter, very high or very low τ values that cannot be fully covered by the applied frequency range, or by the non-semicircular shape of the impedance spectra (Fig. 4A). In these cases, R^epi^ values estimated by the three methods applied in the present study may vary considerably. The resulting errors are especially critical in experiments during which drugs such as forskolin or nystatin had been applied prior to the application of EGTA, as here R^trans^ in the absence of the drugs has to be estimated sequentially from the data obtained in the presence of the drugs (compare Fig. 4B and C).

Whereas R^trans^ values estimated from experiments without drug application were similar for all three methods, data from experiments with drug application were most consistent for ANN evaluation. Most importantly, R^trans^ values from four experiments had to be omitted from evaluation, as they yielded either negative or unreasonably high R^trans^ values (>10^5^ Ω·cm^2^) when evaluated with methods M1 and M2, whereas ANN evaluation was fully within the range of all other experiments (Fig. 4D, evaluation including all experiments; Fig. 4E evaluation omitting four experiments).

**Figure 5:**
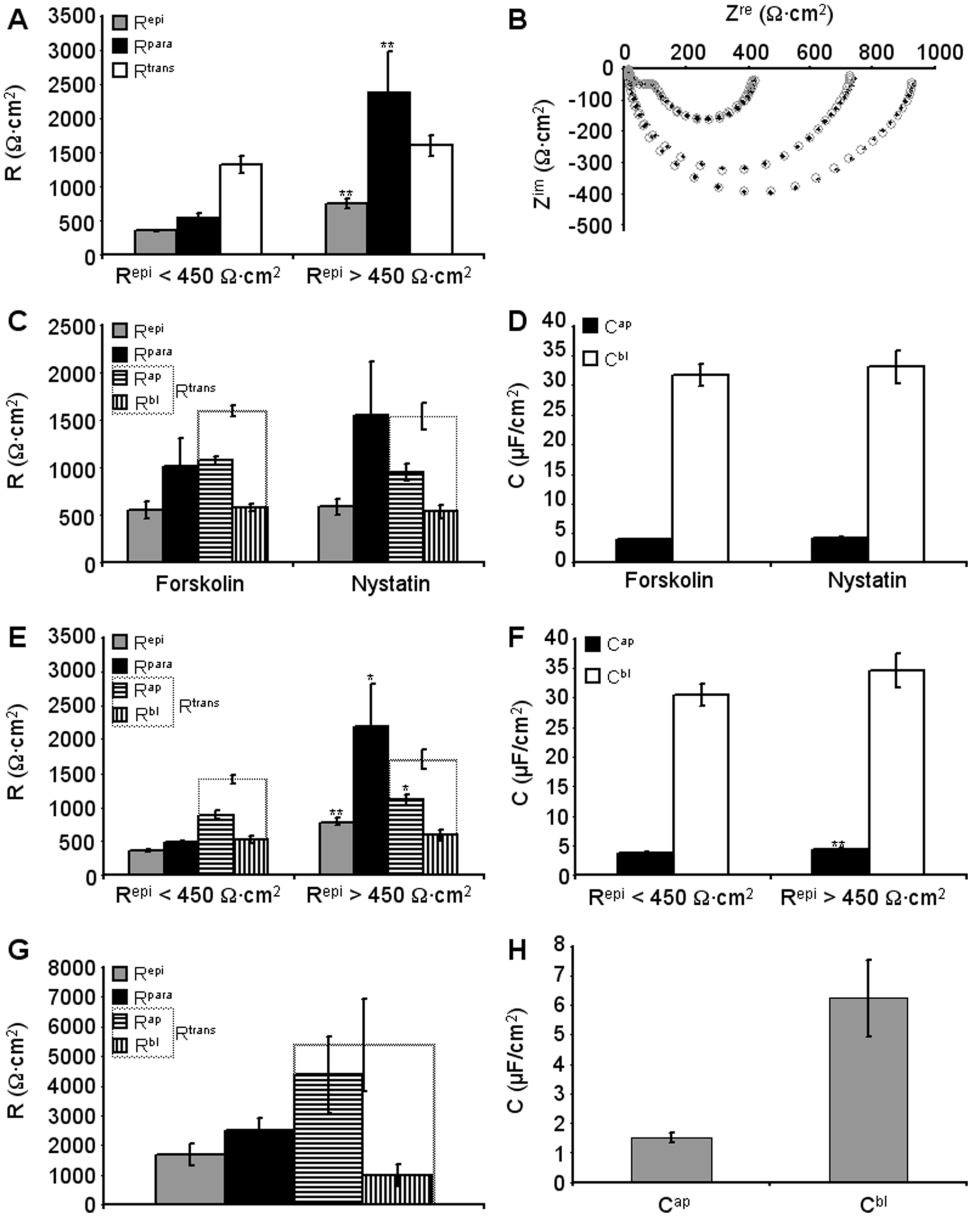
Estimation of apical and basolateral parameters. **A)** HT-29/B6 cell layers were divided in two groups, with R^epi^ values <450 Ω·cm^2^ (354±16 Ω·cm^2^, n = 12) and R^epi^ values >450 Ω·cm^2^ (757±65 Ω·cm^2^, n = 14), respectively. These two groups differed significantly in R^para^ (p<0.01, Student’s t-test) but not in R^trans^. **B)** Set of four impedance spectra obtained in the absence (two larger curves) or presence of forskolin (two smaller curves). Experimental data (♦) were fitted using the six component circuit (○) as described in the methods section, to obtain R^ap^, R^bl^, C^ap^ and C^bl^. **C,D)** R^epi^, R^para^, R^trans^, R^ap^, R^bl^ (C) and C^ap^, C^bl^ (D) values from 11 experiments with apical or baslolateral nystatin application and 7 experiments with forskolin application. For forskolin purely apical effects were assumed. None of the parameters differed significantly between the two groups. **E,F)** Same data as in C and D, but regrouped for R^epi^ <450 Ω·cm^2^ (9 values) and R^epi^ >450 Ω·cm^2^ (9 values). As in (A), R^para^ (p<0.05, Student’s t-test) but not R^trans^ was significantly different between these two groups. However, both, R^ap^ and C^ap^ were significantly larger in cell layers with R^epi^ >450 Ω·cm^2^ (p<0.01, Student’s t-test). **G, H)** R^epi^, R^para^, R^trans^, R^ap^ and R^bl^ (G), and C^ap^ and C^bl^ (H) values for three IPEC-J2 cell layers treated with nystatin.

With increasing number of passages of HT-29/B6 cells, R^epi^ values tend to decrease. To determine whether this decrease is due to changes in R^trans^ or R^epi^, experiments were divided into two groups according to the R^epi^ values of the employed cell layers (R^epi^ <450 Ω·cm^2^, n = 12; R^epi^ >450 Ω·cm^2^, n = 14, boundary arbitrary). As shown in Fig. 5A, differences in R^epi^ are solely due to changes in R^para^ (p<0.01, Student’s t-test), whereas R^trans^ remains unaltered.

### Calculation of R^ap^, R^bl^, C^ap^ and C^bl^

R^para^ values estimated by ANN-enhanced 2PI were used to obtain R^ap^, R^bl^, C^ap^ and C^bl^ by a least*-*squares approach. A direct comparison of measured and fitted impedance values is shown in Fig. 5B. 11 experiments employing nystatin application (5 apical, 6 basolateral) and seven experiments employing forskolin were evaluated assuming that forskolin only affected R^ap^. The values obtained from these latter experiments (Table 2) were not significantly different from the values obtained with nystatin so that it is concluded that the predominant effect of forskolin is a dramatic reduction of R^ap^ (Fig. 5C,D).

**Table 2:** Parameters estimated for HT-29/B6 and IPEC-J2 cells.

	n	R^sub^ [Ω·cm^2^]	R^para^ [Ω·cm^2^]	R^ap^ [Ω·cm^2^]	R^bl^ [Ω·cm^2^]	C^ap^ [µF/cm^2^]	C^bl^ [µF/cm^2^]
**HT-29/B6**							
prior to nystatin	11	13.2±0.6	1552±564	948±88	542±68	4.23±0.12	33.1±2.7
nystatin apical	5			230±15			
nystatin basolateral	6				32±6		
prior to forskolin	7	10.5±1.1	1014±297	1069±47	579±39	3.98±0.12	31.7±1.9
forskolin present	7			89.4±3.3			
Lumped, R^epi^ >450	9	11.8±0.8	2201±608	1106±86	586±75	4.4±0.1	34.7±2.9
Lumped, R^epi^ <450	9	12.5±1.0	485±25	885±59	527±46	3.87±0.08	30.5±1.9
**IPEC-J2**							
	3	9.7±2.0	2508±424	4387±1291	979±360	1.51±0.16	6.24±1.28

Lumping this total of 18 experiments and splitting them into two halfs according to the R^epi^ value (R^epi^ >450 Ω·cm^2^, n = 9, mean R^epi^, 787±55 Ω·cm^2^ vs. R^epi^ <450 Ω·cm^2^, n = 9, mean R^epi^, 359±16 Ω·cm^2^) again indicated that the predominant reason for the reduced R^epi^ values at higher passages was a strong reduction in R^para^ (p<0.05, Fig. 5E), essentially turning the tight epithelium (R^para^>R^trans^) into a leaky one (R^para^<R^trans^). However, small but significant changes could also be detected for R^ap^ (p<0.05) and C^ap^ (p<0.01, Table 2, Fig. 5E,F) which are consistent with a loss of differentiation with increasing numbers of passages.

Repeating the evaluation with R^para^ values obtained through methods M1 and M2 essentially yielded similar results. However, using R^para^ values obtained through ANN produced lowest mean deviation per impedance value in 9 out of the 18 experiments (M1, 5; and M2, 4 of the 18 experiments). It is therefore concluded that values obtained through ANN are more accurate than those obtained through M1 and M2.

For IPEC-J2 cells, main difficulty was that due to their very high R^epi^ values (range 1000 to 10 000 Ω·cm^2^), reliable fluorescein flux measurements were only possible for cell layers with lowest R^epi^ values. Thus, results for three cell layers with a mean R^epi^ of 1692±368 Ω·cm^2^ are shown (Table 2; Fig. 5G,H).

## Discussion

### Neural Network Training and Error Model

Unlike ANN applications based on real-world training data, the present approach of employing modeled impedance spectra provides direct control over sampling and balance of the training data. While it is desirable to avoid imbalanced training data [27], at the same time the so-called “curse of dimensionality” [28] implies a very practical limitation for generating equally-distributed data from more than one input variable [29]; i.e. a set of impedance spectra comprehensively covering all six, or five respectively, circuit parameters would exhibit a size exceeding common training data sizes by orders of ten. Consequently, a bias in either the input or target domain is unavoidable in practice. By backward-calculating the six circuit parameters from the given target value, our approach aims at a balanced target domain with potentially imbalanced training input rather than a relatively balanced training input with an imbalanced target domain.

No negative impact on the ANN training was observed from adding data scatter modeled from the electrophysiological setup; in fact, it is even likely that introducing error to training data is advantageous for the generalization ability of a network [30]–[32]. Modeling such data scatter as standard deviations depending on frequency and R^T^ is a concept that has been successfully applied before [26]. Naturally, the proposed regressions are setup-specific and the implementation is limited to the given settings. This is not considered a limitation of the proposed method, as data scatter intrinsic to any other set-up can be modeled analogously.

For all ANNs, a network structure with a single hidden layer consisting of two units was chosen. While this has previously been shown to be sufficient to approximate any mathematical function [33] and smaller ANNs tend to generalize better [34], [35], this architecture also showed the best learning progress of several architectures initially investigated here by trial-and-error. An “early stopping” strategy (cf. e.g. [36]) at a fixed number of epochs was chosen for the training, as no specific final training error was intended. Overfitting was not observed from average training and validation error (cross validation; cf. e.g. [37]). As previously reported by others [38], [39], trained ANNs were not able to extrapolate target parameters notably beyond the ranges given by the training data.

### Comparison of Modeled and Measured Impedance Spectra

Accuracy of R^epi^ estimates from measured spectra is difficult to assess, as results cannot be tested against “true” values. While it might be argued that true R^T^ can be obtained from DC measurements, most equipment carries out “near DC” measurements at frequencies of about 25 instead of 0 Hz or uses brief current pulses to avoid electrode polarization. In the spectrum depicted in Fig. 4A, the estimated R^T^ is about 600 Ω·cm^2^, however, at 25 Hz (14^th^ data point from the right), a value of about 125 Ω·cm^2^ would have been obtained. Furthermore, to obtain R^epi^, R^sub^ has to be determined first. While this may be comparatively simple for cultured cells grown on filter supports, it is impossible for native tissues without employing impedance spectroscopy [40]. In the present study, the finding that reference areas defined by the data points from model spectra cover most of the data points from measured spectra (Fig. 2C, Fig. S7) is taken as a strong indication that the ANN were trained for appropriate ranges and that predictions for both modeled and measured spectra are more accurate than those from conventional methods. This conclusion is supported by two further findings: (1) In contrast to R^trans^ calculations based on the ANN approach, calculations based on method M1 or M2 led to obviously erroneous R^trans^ values for four out of 30 experiments (cf Fig. 4D). (2) For experiments with and without application of forskolin or nystatin, use of R^epi^ values estimated by ANN resulted in consistent R^trans^ values, whereas especially those determined by method M2 (Cole-Cole-fit) did not. This is due to the fact that Cole-Cole-fits are based on semicircular impedance spectra, a requirement that is not fulfilled after the application of forskolin or nystatin.

### Estimation of Apical and Basolateral Membrane Properties

Testing the reliability of the least*-*squares approach with model spectra yielded average deviations of less than 0.1% (R^ap^, 0.024±0.019%; R^bl^, −0.017±0.057%; C^ap^, −0.047±0.051; C^bl^, 0.039±0.093%; n = 6) if the correct R^para^ was known. 10% variations in R^para^ induced errors <10% in all other parameters if apical nystatin applications were simulated (<15% for basolateral applications). Under experimental conditions target values are unknown and therefore accuracy can only be evaluated indirectly. This was achieved by using the average deviation per data point between measured and modeled impedances. In 9 out of 18 experiments, deviations were lowest when ANNs were employed (5 out of 11 experiments employing nystatin and 4 out of 7 employing forskolin), but only in 5 experiments when using method M1 (4 for nystatin, 1 for forskolin) and 4 when using method M2 (2 for nystatin, 2 for forskolin). This indicates that under both conditions estimates based on ANN evaluation are the most reliable.

R^ap^ values found for HT-29/B6 cells are in a similar range as those published for various preparations, whereas R^bl^ values are considerably higher (*Necturus* gallbladder, R^ap^ 1220 Ω·cm^2^, R^bl^ 201 Ω·cm^2^, [41]; R^ap^ 3500 Ω·cm^2^, R^bl^ 225 Ω·cm^2^, [15]; 2F3 cells, R^ap^, 1270 Ω·cm^2^, R^bl^ 330 Ω·cm^2^ and A6 cells, R^ap^ 2700 Ω·cm^2^, R^bl^ 330 Ω·cm^2^, [42]). However, a high R^bl^ value in HT-29/B6 cells is in keeping with experimental observations upon nystatin application. Basolateral application of nystatin caused a R^epi^ decrease from 524±75 Ω·cm^2^ to 397±52 Ω·cm^2^ (n = 6), whereas apical nystatin application caused a decrease from 586±112 Ω·cm^2^ to 335±61 Ω·cm^2^ (n = 6). Thus, in both cases the residual R^epi^ was similar.

R^trans^ (R^ap^+R^bl^ = 5366 Ω·cm^2^) and R^para^ (2508 Ω·cm^2^) values for IPEC cells are remarkable, as, despite their large R^epi^, these cell layers are “leaky” according to the definition by Schultz et al., [43] (R^para^<R^trans^). It has to be kept in mind, however, that only IPEC-J2 layers with lowest R^epi^ could be analyzed.

Capacitance of unit plasma membranes is about 1 µF/cm^2^ [44], and values for C^ap^ and C^bl^ are often used as direct measures of apical and basolateral membrane areas [13], [16]. Values derived in the present study are in the same range as those previously reported [12], [15], [41], [45]. As Schifferdecker & Frömter [16] point out, a C^ap^/C^bl^ ratio of 1 : 5 would be expected if epithelial cells were perfect cubes joined along their apical-most edge. In the present study, ratios of 1 : 7.9 (±0.4, n = 18) and 1 : 4.4 (±1.4, n = 3) were found for HT-29/B6 and IPEC-J2 cells, respectively. These values indicate that HT-29/B6 cells are columnar whereas IPEC-J2 cells are squamous. This is in keeping with published observations (HT-29/B6, diameter ∼6 µm, height 20–30 µm, [7], [46]; IPEC-J2, diameter ∼13 µm, height ∼7 µm, [47]). Deviations from theoretical C^ap^/C^bl^ values calculated from their dimensions (HT-29/B6, ∼1 : 15 to 20; IPEC-J2, ∼1 : 2) may be explained by abundant microvilli in HT-29/B6 cells [46] and possibly by basolateral membrane foldings in IPEC-J2 cells. It can further be speculated that the increased C^ap^ found in HT-29/B6 cell layers with high R^epi^ indicate a higher degree of differentiation with an even greater abundance of microvilli. The parallel increase in R^ap^ could then simply reflect a high access resistances of these microvilli.

### Effects of Forskolin

Forskolin is known to increase intracellular cAMP levels through activation of the adenylate cyclase [48]. This causes vesicles containing CFTR to merge with the apical membrane which induces Cl^−^ secretion. Forskolin application thus mimics secretory diarrhea, a common intestinal disorder driven by increased chloride secretion. Capacitance changes of the apical membrane during CFTR insertion have been studied in detail by Bertrand et al. [45] and were demonstrated to last for about 10 minutes. Therefore, in the present study, only spectra determined at least 10 minutes after forskolin application were considered. Under these conditions, results were consistent with the assumption that the R^epi^ decrease observed upon forskolin application is primarily due to a decrease in R^ap^.

### Conclusions

The aim of the present study was to develop a technique to quantify partial resistances and capacitances of the overall epithelial barrier of HT-29/B6 and IPEC-J2 cell lines. The employed ANNs permitted reliable extraction of R^sub^ and R^epi^ with higher precision than conventional methods. The use of ANNs thus resulted in a powerful refinement of two-path impedance spectroscopy for cell layers with modified R^ap^ or R^bl^. With this refinement, all six parameters of the equivalent circuit for epithelial cell layers can be estimated for HT-29/B6 and IPEC-J2 cells without using invasive techniques such as intracellular microelectrode recordings. Successful quantification of the effect of forskolin suggests that this technique qualifies as a valuable tool for pharmacological studies during which apical or basolateral membrane properties are altered.

## Supporting Information

Figure S1 **Nyquist diagram of HT-29/B6 impedance spectra.**
(PDF)

Figure S2 **Setup-specific data scatter.**
(PDF)

Figure S3 **ANN architecture.**
(PDF)

Figure S4 **ANN training progress.**
(PDF)

Figure S5 **Comparison of M1, M2 and ANN performance on modeled impedance spectra.**
(PDF)

Figure S6 **Similarity diagrams comparing M1, M2 and ANN performance on modeled vs. experimental impedance spectra.**
(PDF)

Figure S7 **Evaluation of similarity diagrams.**
(PDF)

Table S1 **Characteristics of modeled datasets.**
(PDF)

Table S2 **Characteristics of datasets used for training ANNs to estimate epithelial resistance (R^epi^).**
(PDF)

Table S3 **Characteristics of datasets used for training ANNs to estimate subepithelial resistance (R^sub^).**
(PDF)

Table S4 **Error of test data for all 8 employed neural networks, determined after ANN training was completed. For a performance comparision with reference methods M1 and M2 see Fig. 5a and 5b.**
(PDF)

Table S5 **Characteristics of measured datasets.**
(PDF)

File S1 **Real (Z^re^) and imaginary (Z^im^) part of the complex total impedance Z^T^; Eq. S1–Eq. S5.**
(PDF)

File S2 **Impact of inaccurate determination of R^sub^ and R^epi^; Eq. S6–Eq. S13.**
(PDF)

File S3 **Datasets overview: preprocessing, benchmarking data, frequencies.**
(PDF)

File S4 **Error model; Eq. S14–Eq. S17.**
(PDF)
