# Supplementary material for: Discerning Apical and Basolateral Properties of HT-29/B6 and IPEC-J2 Cell Layers by Impedance Spectroscopy, Mathematical Modeling and Machine Learning
Source: PLoS One. 2013 Jul 1;8(7):e62913. doi: 10.1371/journal.pone.0062913 (PMC3698131; doi:10.1371/journal.pone.0062913)
Supplement: Figure S1 — Nyquist diagram of HT-29/B6 impedance spectra. (PDF) [file pone.0062913.s001.pdf]

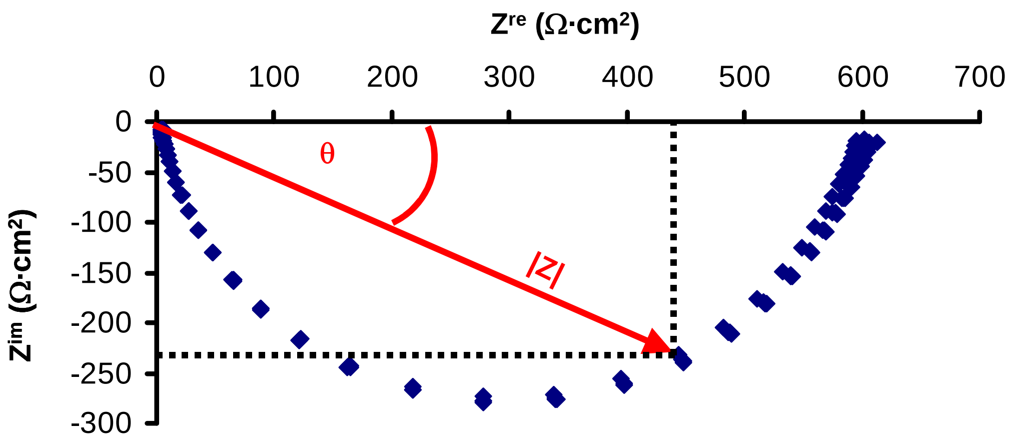

Fig. S1: Nyquist diagram of HT-29/B6 impedance spectra

Four consecutive impedance spectra of an HT-29/B6 cell layer mounted in an Ussing chamber were recorded at 42 different frequencies, f (◆). As an example, impedance magnitude |Z| and phase angle θ between transepithelial alternating current (AC) and voltage are depicted for f ≈ 33 Hz (red arrow). Real (Z^{re}) and imaginary part (Z^{im}) of the complex impedance (total impedance $Z^T(f) = Z^{re}(f) + i \cdot Z^{im}(f)$, $i = \sqrt{-1}$) are the projections onto the x- and y-axis.

For f → 0 (DC conditions), θ → 0°. For f →∞, θ → 90°.
