# Supplementary material for: Discerning Apical and Basolateral Properties of HT-29/B6 and IPEC-J2 Cell Layers by Impedance Spectroscopy, Mathematical Modeling and Machine Learning
Source: PLoS One. 2013 Jul 1;8(7):e62913. doi: 10.1371/journal.pone.0062913 (PMC3698131; doi:10.1371/journal.pone.0062913)
Supplement: Figure S2 — Setup-specific data scatter. (PDF) [file pone.0062913.s002.pdf]

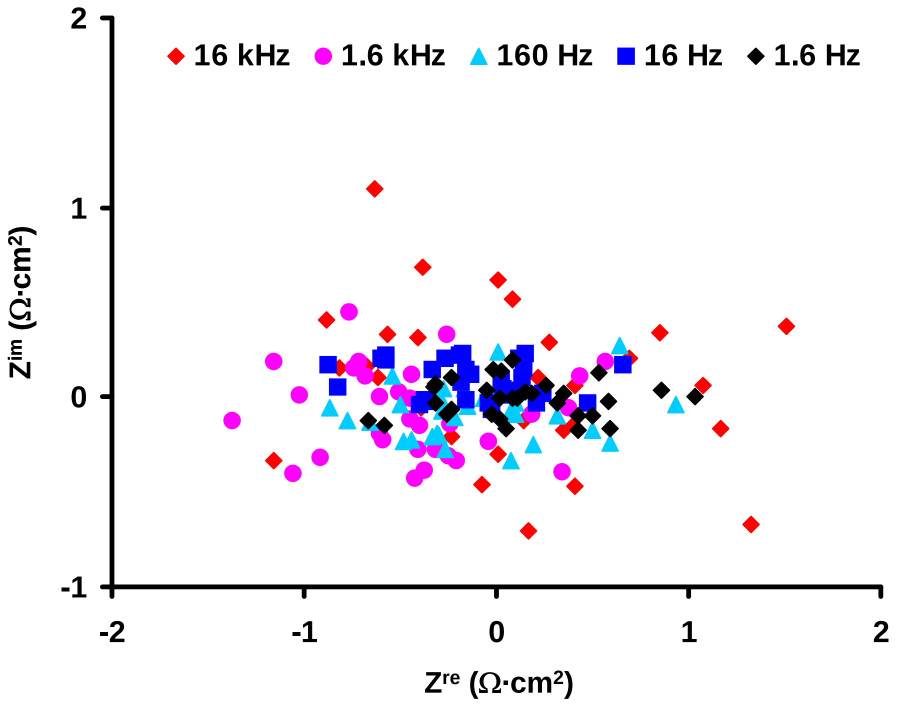

Fig. S2: Setup-specific data scatter

Impedances of an artificial membrane mounted in an Ussing chamber ($R^T \approx 500\ \Omega \cdot cm^2$) were repeatedly recorded at 42 different frequencies, f. As an example, values recorded at five different frequencies (f ≈ 1.6, 16, 160, 1600, and 16000 Hz) are shown. Scatter of Z^{re} and Z^{im} increased with increasing f and with increasing R^T of the artifiicial membrane (not shown).
