# Supplementary material for: Discerning Apical and Basolateral Properties of HT-29/B6 and IPEC-J2 Cell Layers by Impedance Spectroscopy, Mathematical Modeling and Machine Learning
Source: PLoS One. 2013 Jul 1;8(7):e62913. doi: 10.1371/journal.pone.0062913 (PMC3698131; doi:10.1371/journal.pone.0062913)
Supplement: Figure S3 — ANN architecture. (PDF) [file pone.0062913.s003.pdf]

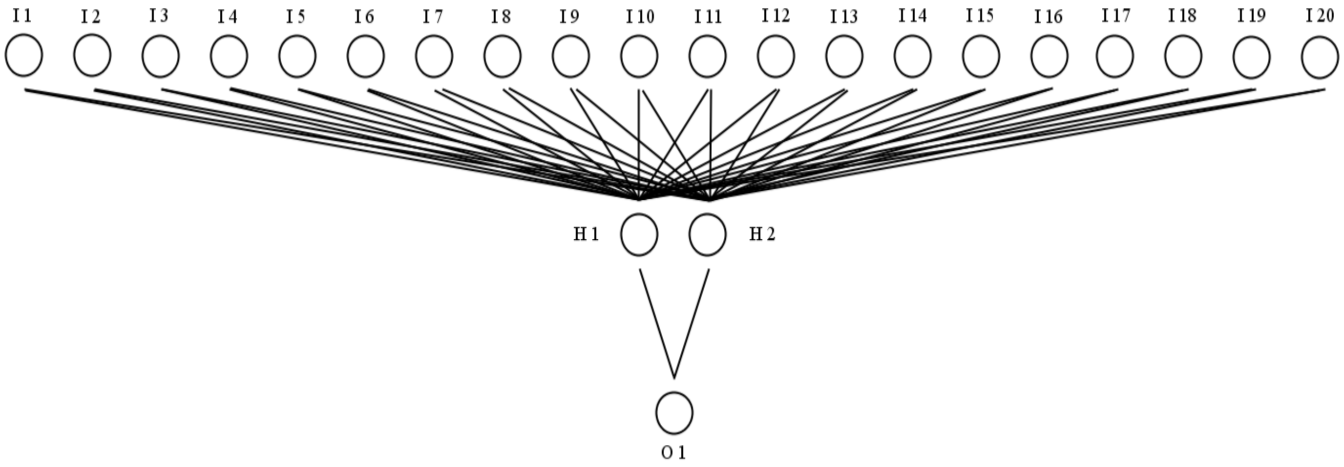

Fig. S3: ANN architecture
Graphical interpretation of a 20-2-1 feed-forward ANN architecture as used in the present study for all networks. Each node represents a network unit consisting of an activation function, each line represents a weighted unidirectional connection between function outputs and function inputs. Units I1 to I20 represent the input layer of the network, units H1 and H2 represent the hidden layer of the network, and unit O1 represents the output layer of the network. Activation functions of all input and output units are linear, f(x)=x with range [-∞,∞] , while activation functions of all hidden units are sigmoidal, $f(x)= 1/(1+e^{-x})–½$ with range [-0.5, 0.5]. All ANN were simulated with the FORWISS Artificial Neural Network Simulation Toolbox (FAST) v2.2 (for documentation cf. Arras and Mohraz, 1996).

References

Arras, M. K., K. Mohraz. 1996. FORWISS Artificial Neural Network Simulation Toolbox (FAST) v2.2, Bavarian Research Center for Knowledge-Based Systems, FORWISS, Erlangen, Germany.
