# Supplementary material for: Discerning Apical and Basolateral Properties of HT-29/B6 and IPEC-J2 Cell Layers by Impedance Spectroscopy, Mathematical Modeling and Machine Learning
Source: PLoS One. 2013 Jul 1;8(7):e62913. doi: 10.1371/journal.pone.0062913 (PMC3698131; doi:10.1371/journal.pone.0062913)
Supplement: Figure S4 — ANN training progress. (PDF) [file pone.0062913.s004.pdf]

ANN_{sub}^{HT}

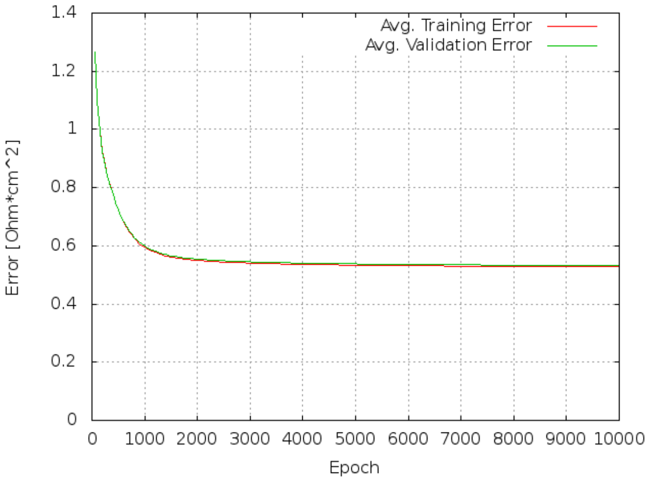

$ANN_{sub}^{HT+EGTA}$

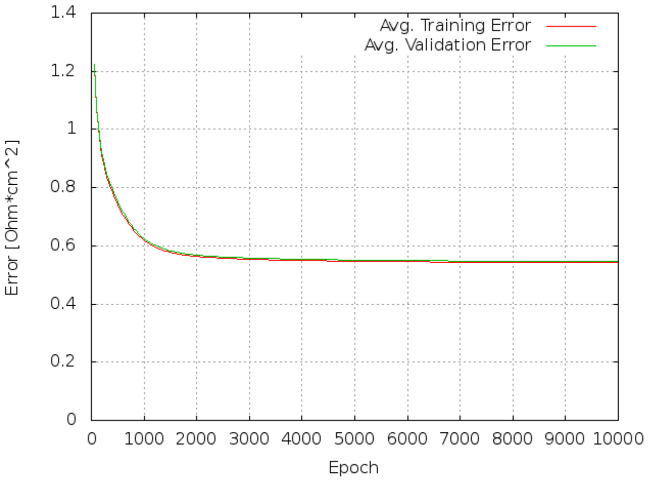

ANN_{epi}^{HT}

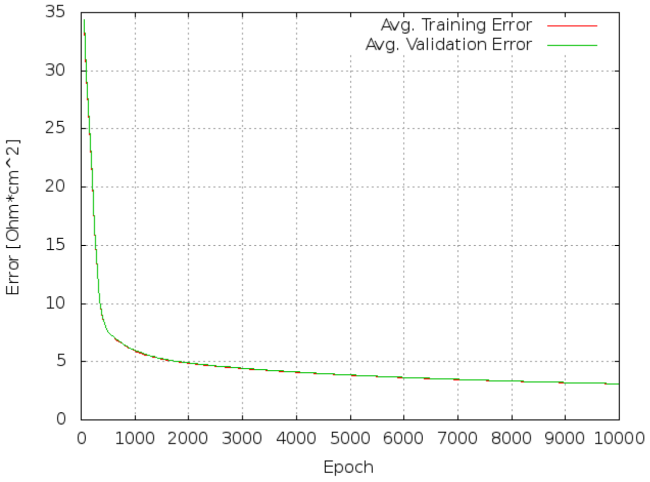

$ANN_{epi}^{HT+EGTA}$

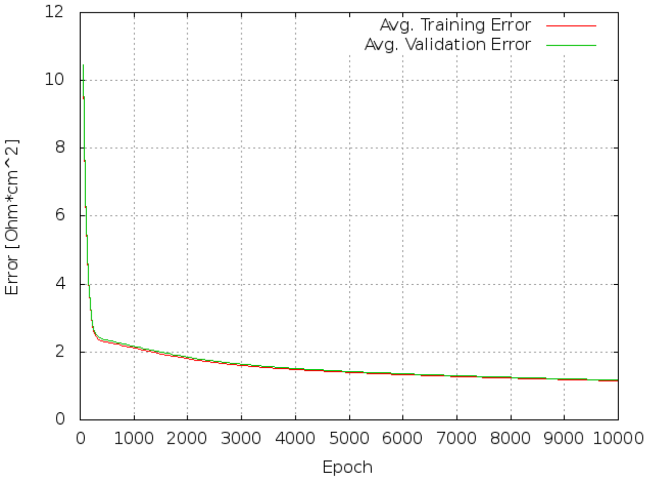

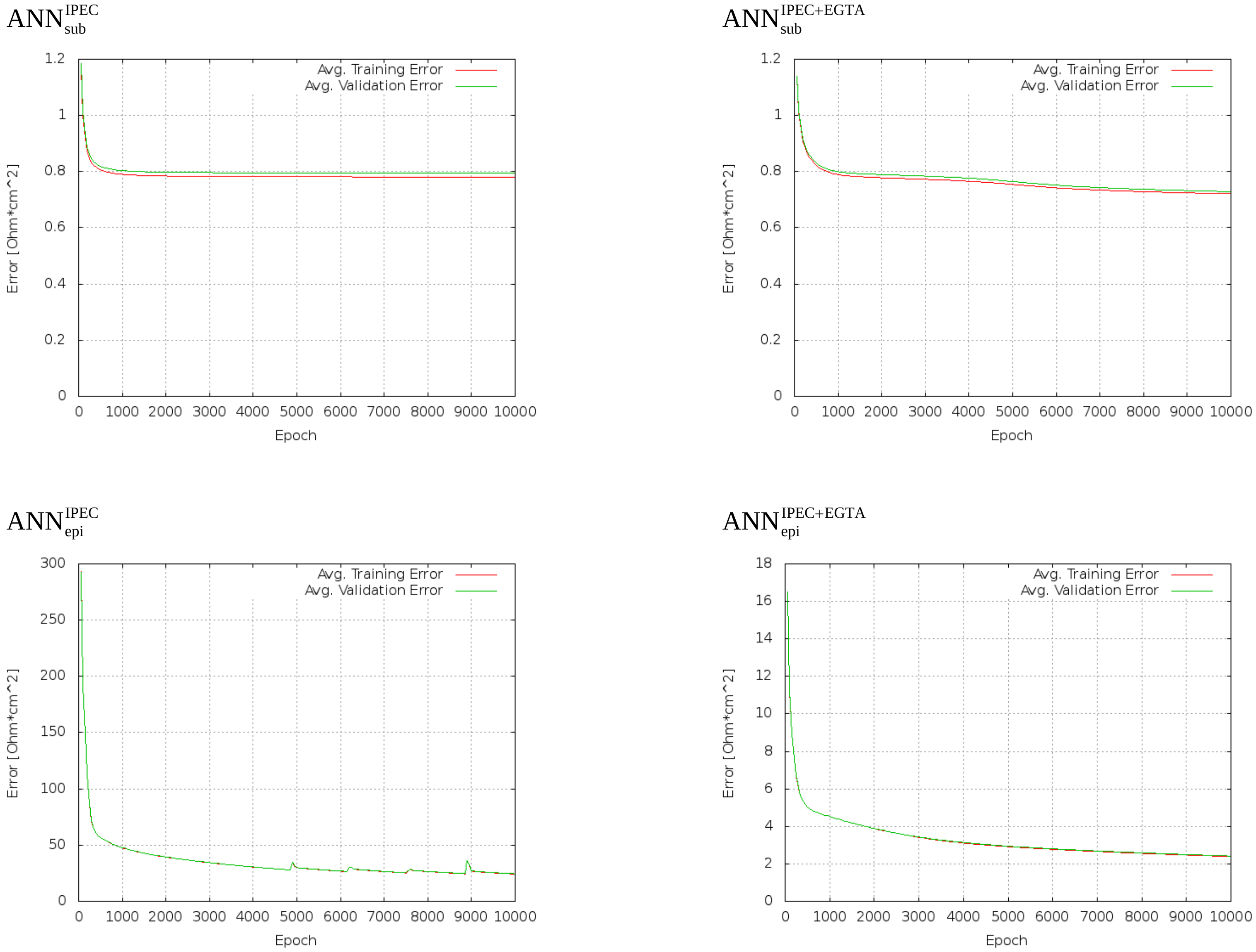

Fig. S4. ANN training progress

Development of average deviations from target values of training and validation data during 10,000 training epochs. For results see Table S4.
