# Supplementary material for: Discerning Apical and Basolateral Properties of HT-29/B6 and IPEC-J2 Cell Layers by Impedance Spectroscopy, Mathematical Modeling and Machine Learning
Source: PLoS One. 2013 Jul 1;8(7):e62913. doi: 10.1371/journal.pone.0062913 (PMC3698131; doi:10.1371/journal.pone.0062913)
Supplement: Figure S5 — Comparison of M1, M2 and ANN performance on modeled impedance spectra. (PDF) [file pone.0062913.s005.pdf]

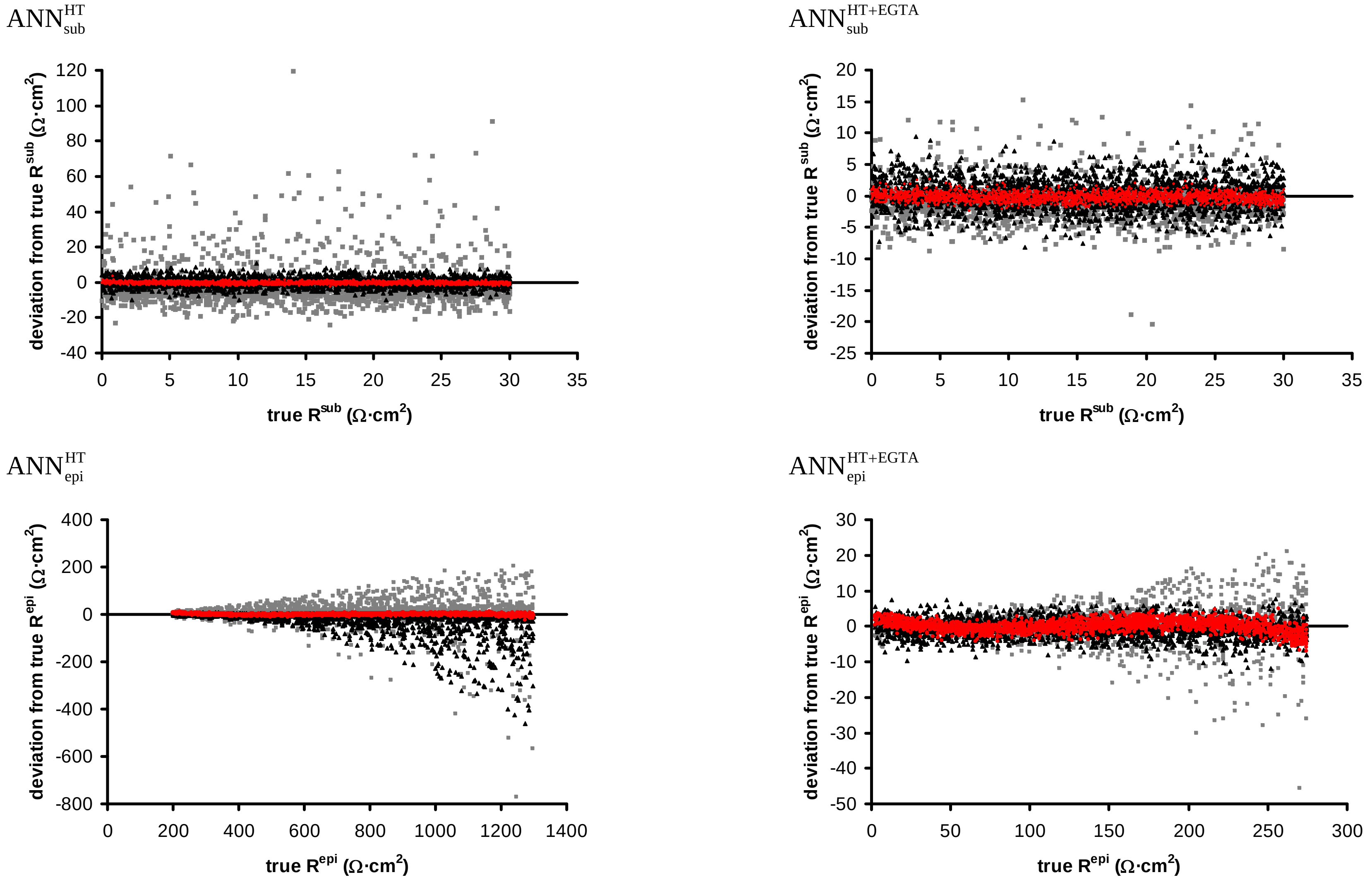

ANN_{sub}^{HT}
deviation from true R^{sub} ($\Omega\cdot cm^2$)
true R^{sub} ($\Omega\cdot cm^2$)
$ANN_{sub}^{HT+EGTA}$
deviation from true R^{sub} ($\Omega\cdot cm^2$)
true R^{sub} ($\Omega\cdot cm^2$)
ANN_{epi}^{HT}
deviation from true R^{epi} ($\Omega\cdot cm^2$)
true R^{epi} ($\Omega\cdot cm^2$)
$ANN_{epi}^{HT+EGTA}$
deviation from true R^{epi} ($\Omega\cdot cm^2$)
true R^{epi} ($\Omega\cdot cm^2$)

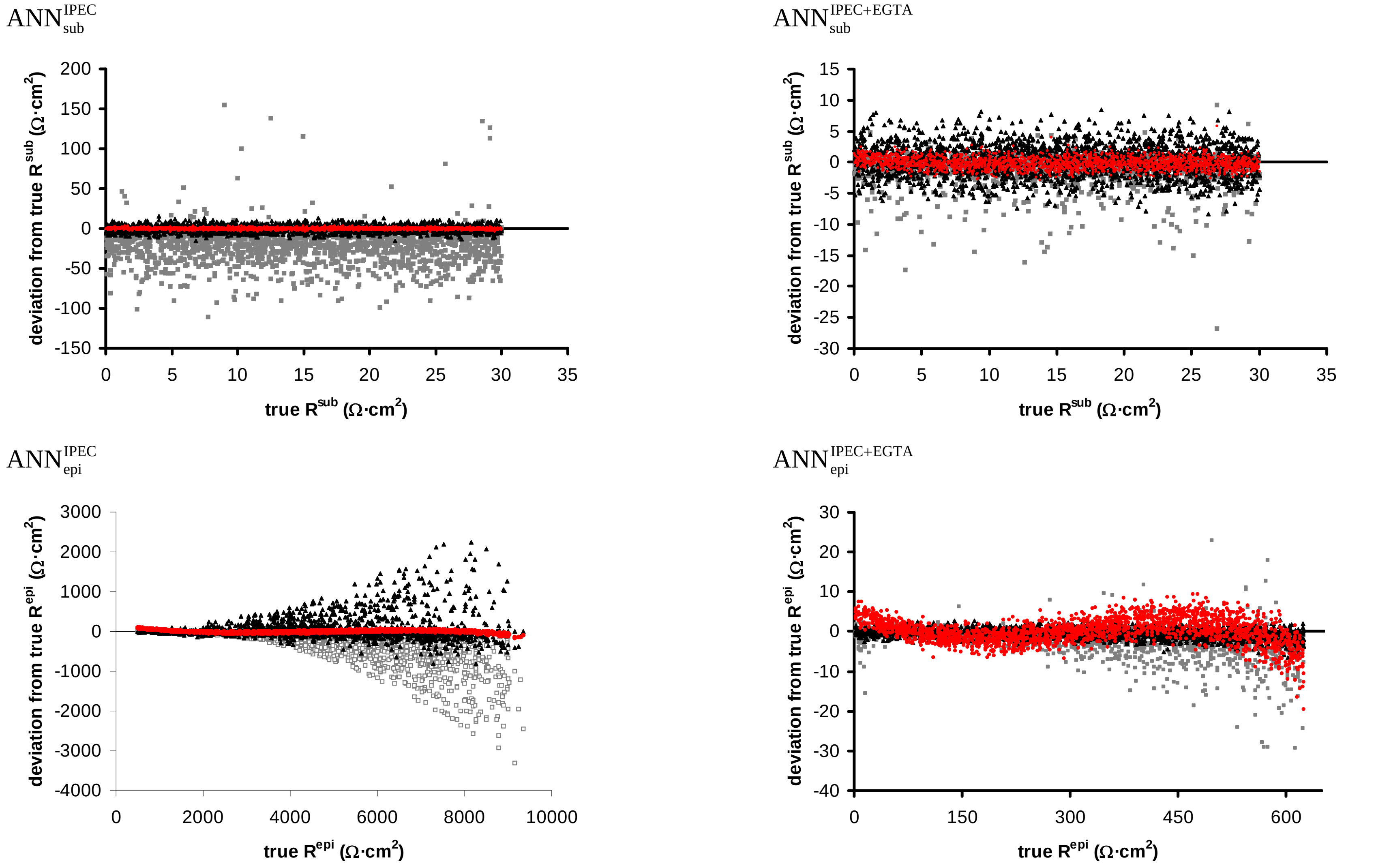

Fig. S5: Comparison of M1, M2 and ANN performance on modeled impedance spectra

Deviations of estimated R^{epi} and R^{sub} values (estimated minus true value), using method M1 (▲), M2(■) and the indicated ANN (●) are plotted against the respective true value. ANN perform better that M1 and M2 in all cases except $ANN_{epi}^{IPEC+EGTA}$.
