# Supplementary material for: Discerning Apical and Basolateral Properties of HT-29/B6 and IPEC-J2 Cell Layers by Impedance Spectroscopy, Mathematical Modeling and Machine Learning
Source: PLoS One. 2013 Jul 1;8(7):e62913. doi: 10.1371/journal.pone.0062913 (PMC3698131; doi:10.1371/journal.pone.0062913)
Supplement: Figure S6 — Similarity diagrams comparing M1, M2 and ANN performance on modeled vs. experimental impedance spectra. (PDF) [file pone.0062913.s006.pdf]

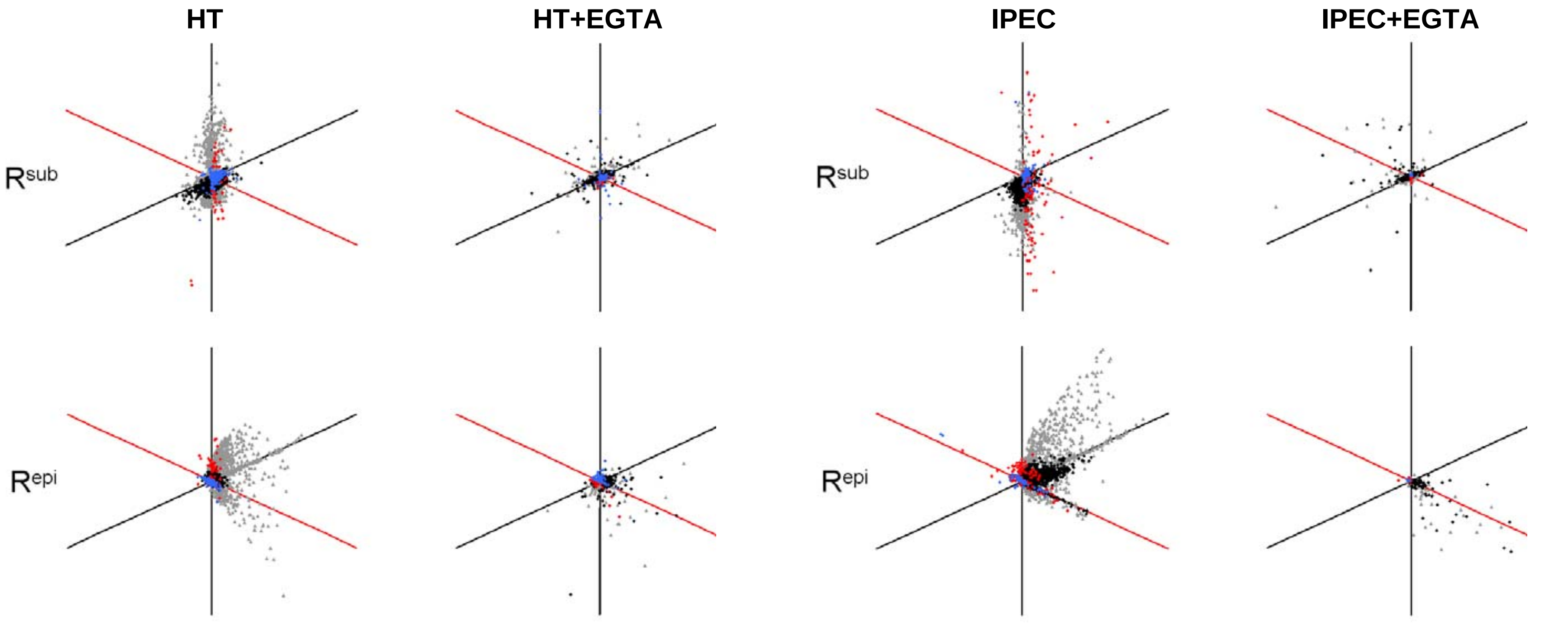

Fig. S6: Similarity diagrams comparing M1, M2 and ANN performance on modeled vs. experimental impedance spectra
Similarity diagrams of data were derived for modeled and measured spectra (HT, IPEC, HT+EGTA, IPEC+EGTA, targeting either R^{epi} or R^{sub} as indicated) as illustrated in Fig. 2 of the main text. Data scatter along the red axis indicates a greater dissimilarity of values obtained with the appropriate ANN when method M1 and M2 yield very similar results.
In all cases, distribution of data points from modeled spectra with τ-ratios < 5 (◆) is similar to the distribution of data points from measured spectra (●) obtained in the absence of forskolin or nystatin. Conversely, distribution of data points from modeled spectra with τ-ratios > 5 (▲) is similar to the distribution of data points from experimental spectra (◆) obtained in the presence of forskolin or nystatin.
