# Supplementary material for: Discerning Apical and Basolateral Properties of HT-29/B6 and IPEC-J2 Cell Layers by Impedance Spectroscopy, Mathematical Modeling and Machine Learning
Source: PLoS One. 2013 Jul 1;8(7):e62913. doi: 10.1371/journal.pone.0062913 (PMC3698131; doi:10.1371/journal.pone.0062913)
Supplement: Figure S7 — Evaluation of similarity diagrams. (PDF) [file pone.0062913.s007.pdf]

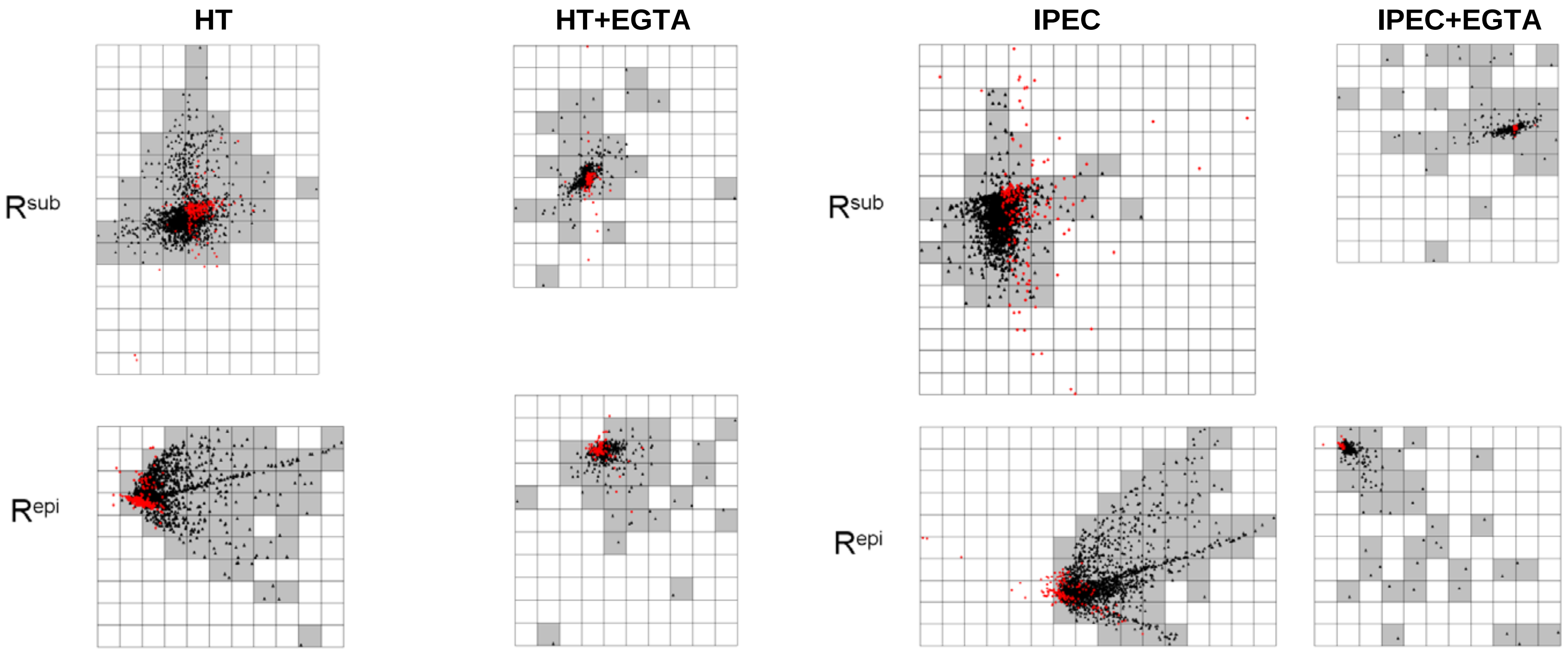

Fig. S7: Evaluation of similarity diagrams

Shaded "reference" areas, indicate areas covered by data points obtained from modeled spectra (▲) within a 10 by 10 grid. Percentage of data points from measured spectra (◆) within the reference areas was evaluated.

Results are given as % (n/m), with m being the total number of data points and n the number of data points within the reference area:

ANN_{sub}^{HT}	98.2% (276/281)	ANN_{sub}^{IPEC}	80.1% (133/166)
$ANN_{sub}^{HT+EGTA}$	97.3% (109/112)	$ANN_{sub}^{IPEC+EGTA}$	100% (26/26)
ANN_{epi}^{HT}	98.2% (276/281)	ANN_{epi}^{IPEC}	82.5% (137/166)
$ANN_{epi}^{HT+EGTA}$	95.5% (107/112)	$ANN_{epi}^{IPEC+EGTA}$	96.2% (25/26)

High percentages indicate that modeled spectra used for ANN training appropriately cover physiologically relevant parameter ranges.
