# Supplementary material for: Discerning Apical and Basolateral Properties of HT-29/B6 and IPEC-J2 Cell Layers by Impedance Spectroscopy, Mathematical Modeling and Machine Learning
Source: PLoS One. 2013 Jul 1;8(7):e62913. doi: 10.1371/journal.pone.0062913 (PMC3698131; doi:10.1371/journal.pone.0062913)
Supplement: Table S1 — Characteristics of modeled datasets. (PDF) [file pone.0062913.s008.pdf]

Table S1: Characteristics of modeled datasets.

	**HT**	**HT+EGTA**	**IPEC**	**IPEC+EGTA**
Number of samples	331,794	167,379	913,521	536,057
Number of features per sample	84	84	84	84
Exact target values known	Yes	Yes	Yes	Yes
Target domain A	0.0	0.0	0.0	0.0
Range of target domain B	200.0 – 1299.5	2.7 – 274.3	500.8 – 9344.2	1.4 – 624.9

Datasets model four different experiment conditions (HT and IPEC cells before the application of EGTA and in the presence of EGTA). Target domain A refers to subepithelial resistance (R^{sub}), target domain B to epithelial resistance (R^{epi}).
