# Supplementary material for: Discerning Apical and Basolateral Properties of HT-29/B6 and IPEC-J2 Cell Layers by Impedance Spectroscopy, Mathematical Modeling and Machine Learning
Source: PLoS One. 2013 Jul 1;8(7):e62913. doi: 10.1371/journal.pone.0062913 (PMC3698131; doi:10.1371/journal.pone.0062913)
Supplement: Table S2 — Characteristics of datasets used for training ANNs to estimate epithelial resistance (Repi). (PDF) [file pone.0062913.s009.pdf]

Table S2: Characteristics of datasets used for training ANNs to estimate epithelial resistance (R^{epi}).

	**HT**	**HT+EGTA**	**IPEC**	**IPEC+EGTA**
Number of training samples	25,000	25,000	25,000	25,000
Number of test samples	25,000	25,000	25,000	25,000
Number of features per sample	20	20	20	20
Exact target value known	Yes	Yes	Yes	Yes
Range of target domain	200.0 – 1299.5	2.7 – 274.3	500.8 – 9344.2	1.4 – 624.9
Name of trained ANN	ANN_{epi}^{HT}	$ANN_{epi}^{HT+EGTA}$	ANN_{epi}^{IPEC}	$ANN_{epi}^{IPEC+EGTA}$

Datasets were created by random selection from the modeled datasets (Table S1). For use with ANNs only curve features 1-20 (=real and imaginary parts of the impedance values derived from the 10 lowest frequencies) were used. Test samples were also used for validation during training (solely for monitoring learning progress, not for weight adjustment).
