# Supplementary material for: Discerning Apical and Basolateral Properties of HT-29/B6 and IPEC-J2 Cell Layers by Impedance Spectroscopy, Mathematical Modeling and Machine Learning
Source: PLoS One. 2013 Jul 1;8(7):e62913. doi: 10.1371/journal.pone.0062913 (PMC3698131; doi:10.1371/journal.pone.0062913)
Supplement: Table S3 — Characteristics of datasets used for training ANNs to estimate subepithelial resistance (Rsub). (PDF) [file pone.0062913.s010.pdf]

Table S3: Characteristics of datasets used for training ANNs to estimate subepithelial resistance (R^{sub}).

	**HT**	**HT+EGTA**	**IPEC**	**IPEC+EGTA**
Number of training samples	25,000	25,000	25,000	25,000
Number of test samples	25,000	25,000	25,000	25,000
Number of features per sample	20	20	20	20
Exact target value known	Yes	Yes	Yes	Yes
Range of target domain	0.0 – 30.0	0.0 – 30.0	0.0 – 30.0	0.0 – 30.0
Name of trained ANN	ANN_{sub}^{HT}	$ANN_{sub}^{HT+EGTA}$	ANN_{sub}^{IPEC}	$ANN_{sub}^{IPEC+EGTA}$

Datsets were derived from the datasets for training ANNs to estimate R^{epi} (Table S2) by randomly shifting the real parts (=adding a random value matching the range of target domain). For use with ANNs, only curve features 65-84 (=real and imaginary parts of the impedance values derived from the 10 highest frequencies) were used. Test samples were also used for validation during training (solely for monitoring learning progress, not for weight adjustment).
