# Supplementary material for: Discerning Apical and Basolateral Properties of HT-29/B6 and IPEC-J2 Cell Layers by Impedance Spectroscopy, Mathematical Modeling and Machine Learning
Source: PLoS One. 2013 Jul 1;8(7):e62913. doi: 10.1371/journal.pone.0062913 (PMC3698131; doi:10.1371/journal.pone.0062913)
Supplement: Table S4 — Error of test data for all 8 employed neural networks, determined after ANN training was completed. For a performance comparision with reference methods M1 and M2 see Fig. 5a and 5b. (PDF) [file pone.0062913.s011.pdf]

Table S4: Error of test data for all 8 employed neural networks, determined after ANN training was completed. For a performance comparision with reference methods M1 and M2 see Fig. 5a and 5b.

	**Average error** [Ω·cm^2]	**Minimum error** [Ω·cm^2]	**Maximum error** [Ω·cm^2]
**HT-29/B6**			
ANN_{sub}^{HT}	±0.53	-3.40	+2.75
$ANN_{sub}^{HT+EGTA}$	±0.55	-3.04	+2.57
ANN_{epi}^{HT}	±3.11	-14.17	+34.84
$ANN_{epi}^{HT+EGTA}$	±1.17	-5.54	+11.36
**IPEC-J2**			
ANN_{sub}^{IPEC}	±0.79	-4.38	+4.81
$ANN_{sub}^{IPEC+EGTA}$	±0.73	-5.78	3.55
ANN_{epi}^{IPEC}	±24.52	-98.88	+192.93
$ANN_{epi}^{IPEC+EGTA}$	±2.42	-10.04	+19.96
