# Supplementary material for: Discerning Apical and Basolateral Properties of HT-29/B6 and IPEC-J2 Cell Layers by Impedance Spectroscopy, Mathematical Modeling and Machine Learning
Source: PLoS One. 2013 Jul 1;8(7):e62913. doi: 10.1371/journal.pone.0062913 (PMC3698131; doi:10.1371/journal.pone.0062913)
Supplement: Table S5 — Characteristics of measured datasets. (PDF) [file pone.0062913.s012.pdf]

Table S5: Characteristics of measured datasets.

	**HT**	**HT+EGTA**	**IPEC**	**IPEC+EGTA**
Number of samples	281	112	166	26
Number of features per sample	84	84	84	84
Exact target values known	No	No	No	No
Range of target domain A (M1)	2.1 – 28	0.8 – 29	-17 – 44	-10 – 20
Range of target domain A (M2)	-33 – 35	-0.3 – 34	-66 – 89	-15 – 13
Range of target domain B (M1)	231 – 1228	7 – 263	640 – 10962	26 – 586
Range of target domain B (M2)	250 – 1233	3.5 – 250	623 – 11026	26 – 608

Target domain A refers to subepithelial resistance (R^{sub}), target domain B to epithelial resistance (R^{epi}). Range of target domains approximated using reference methods M1 and M2 (cf. methods and materials section).
