# Supplementary material for: Discerning Apical and Basolateral Properties of HT-29/B6 and IPEC-J2 Cell Layers by Impedance Spectroscopy, Mathematical Modeling and Machine Learning
Source: PLoS One. 2013 Jul 1;8(7):e62913. doi: 10.1371/journal.pone.0062913 (PMC3698131; doi:10.1371/journal.pone.0062913)
Supplement: File S1 — Real (Zre) and imaginary (Zim) part of the complex total impedance ZT; Eq. S1–Eq. S5. (PDF) [file pone.0062913.s013.pdf]

Real (Z^{re}) and imaginary (Z^{im}) part of the complex total impedance Z^T

The derivation of the real (Z^{re}) and imaginary (Z^{im}) part of the complex total impedance, Z^T, has recently been revisited (Krug et al, 2009) and is briefly explained here for the three parameter model depicted in the main text (Fig. 1B). In this model, impedance of the (ohmic) epithelial resistor is R^{epi}, that of the subepithelial resistor is R^{sub}. Impedance of the epithelial capacitor depends on the angular frequency ($\omega = 2\cdot\pi\cdot f$) and amounts to $1/(i\cdot\omega\cdot C^{epi})$ where $i = \sqrt{-1}$ denotes the phase shift by -90° and C^{epi} the epithelial capacitance. Applying Kirchhoff's laws, the total impedance Z^T is calculated as:

$$Z^T = \frac{R^{epi}\cdot\left(1 - i\cdot\omega\cdot R^{epi}\cdot C^{epi}\right)}{1+\left(\omega\cdot R^{epi}\cdot C^{epi}\right)^2} + R^{sub} \qquad \text{(Eq. S1)}$$

Z^T is a complex number and can be rearranged to obtain the form $Z^{re} + i\cdot Z^{im}$ (Z^{re}, real part of the impedance or resistance; Z^{im}, imaginary part of the impedance or reactance):

$$Z^{re} = \frac{R^{epi}}{1+\left(\omega\cdot R^{epi}\cdot C^{epi}\right)^2} + R^{sub}; \qquad Z^{im} = \frac{-\omega\cdot(R^{epi})^2\cdot C^{epi}}{1+\left(\omega\cdot R^{epi}\cdot C^{epi}\right)^2} \qquad \text{(Eqs. S2; S3)}$$

For the six parameter model (main text, Fig. 1D), the derivation can be carried out analogously.

The equation for Z^T is given in the main text (Eq. 6) and can be rearranged to yield

$$Z^{re}(\omega) = \frac{R^{para}\cdot\{(R^{ap}+R^{bl})\cdot(R^{para}+R^{ap}+R^{bl})+\omega^2\cdot[(R^{ap}\cdot\tau^{bl}+R^{bl}\cdot\tau^{ap})^2+R^{para}\cdot(R^{ap}\cdot\tau^{bl^2}+R^{bl}\cdot\tau^{ap^2})]\}}{(R^{para}+R^{ap}+R^{bl}-\omega^2\cdot R^{para}\cdot\tau^{ap}\cdot\tau^{bl})^2+\omega^2\cdot(R^{para}\cdot\tau^{ap}+R^{para}\cdot\tau^{bl}+R^{ap}\cdot\tau^{bl}+R^{bl}\cdot\tau^{ap})^2} + R^{sub} \qquad \text{(Eq. S4)}$$

and

$$Z^{im}(\omega) = \frac{-\omega\cdot(R^{para})^2\cdot\{R^{ap}\cdot\tau^{ap}\cdot[1+(\omega\cdot\tau^{bl})^2]+R^{bl}\cdot\tau^{bl}\cdot[1+(\omega\cdot\tau^{ap})^2]\}}{(R^{para}+R^{ap}+R^{bl}-\omega^2\cdot R^{para}\cdot\tau^{ap}\cdot\tau^{bl})^2+\omega^2\cdot(R^{para}\cdot\tau^{ap}+R^{para}\cdot\tau^{bl}+R^{ap}\cdot\tau^{bl}+R^{bl}\cdot\tau^{ap})^2} \qquad \text{(Eq. S5)}$$

References

Krug, S. M., M. Fromm, and D. Günzel. 2009. Two-path impedance spectroscopy for measuring paracellular and transcellular epithelial resistance. *Biophys. J.* 97:2202–2211.
