# Supplementary material for: Discerning Apical and Basolateral Properties of HT-29/B6 and IPEC-J2 Cell Layers by Impedance Spectroscopy, Mathematical Modeling and Machine Learning
Source: PLoS One. 2013 Jul 1;8(7):e62913. doi: 10.1371/journal.pone.0062913 (PMC3698131; doi:10.1371/journal.pone.0062913)
Supplement: File S2 — Impact of inaccurate determination of Rsub and Repi; Eq. S6–Eq. S13. (PDF) [file pone.0062913.s014.pdf]

Impact of inaccurate determination of R^{sub} and R^{epi}

As described by Krug et al (2009, see also Fig. 4B and C of the present paper), transcellular (R^{trans}) and paracellular (R^{para}) resistances of an epithelial cell layer can be estimated from experiments during which R^{para} is altered (e.g. by Ca^{2+} switch) and the resulting change in paracellular conductance is monitored by measuring changes in the flux of a paracellular marker substance (e.g. fluorescein). Routinely, three values before and at least three values after inducing the Ca^{2+} switch are used for the evaluation of one cell layer.

For simplification, here only one value is considered before (transepithelial resistance, R_1^T; flux J_1) and one value after inducing the Ca^{2+} switch (R_2^T; J_2) are considered during the following calculations. R^T is the sum of the subepithelial and the epithelial resistance ($R^T = R^{sub} + R^{epi}$). X is defined as J_1/J_2 ($= R_2^{para} / R_1^{para}$).

In theory, this allows direct calculation of R_1^{para} from measured parameters:

$$R_1^{para} = \frac{R_1^{epi} \cdot R_2^{epi} \cdot (1 - X)}{X \cdot (R_1^{epi} - R_2^{epi})} \qquad \text{(Eq. S6)}$$

In reality, however, not the true R^{epi}, but only an error-inherent approximation $R^{epi} + \Delta R^{epi}$ can be determined for each measurement with $\Delta R^{epi} = \Delta R^T + \Delta R^{sub}$. For Eq. S6, this implies:

$$R_1^{para\,'} = \frac{(R_1^{epi} + \Delta R_1^{epi}) \cdot (R_2^{epi} + \Delta R_2^{epi}) \cdot (1 - X)}{X \cdot ((R_1^{epi} + \Delta R_1^{epi}) - (R_2^{epi} + \Delta R_2^{epi}))} = \frac{(R_1^{epi} \cdot R_2^{epi}) \cdot (1 - X)}{X \cdot ((R_1^{epi} - R_2^{epi}) + (\Delta R_1^{epi} - \Delta R_2^{epi}))} + \frac{(R_1^{epi} \cdot \Delta R_2^{epi} + R_2^{epi} \cdot \Delta R_1^{epi} + \Delta R_1^{epi} \cdot \Delta R_2^{epi}) \cdot (1 - X)}{X \cdot ((R_1^{epi} - R_2^{epi}) + (\Delta R_1^{epi} - \Delta R_2^{epi}))} \qquad \text{(Eq. S7)}$$

While a multiplicative impact is not apparent in this representation, it becomes more obvious if ΔR_1^{epi} and ΔR_2^{epi} are assumed to be of equal value and a representative variable $\Delta R^{epi} = \Delta R_1^{epi} = \Delta R_2^{epi}$ is introduced. Using this assumption reveals that even small differences in R^{epi} values can lead to considerable differences for R^{para}:

$$R_1^{para\,'} = \frac{R_1^{epi} \cdot R_2^{epi} \cdot (1 - X)}{X \cdot (R_1^{epi} - R_2^{epi})} + \frac{(R_1^{epi} \cdot \Delta R^{epi} + R_2^{epi} \cdot \Delta R^{epi} + (\Delta R^{epi})^2) \cdot (1 - X)}{X \cdot (R_1^{epi} - R_2^{epi})} = \frac{R_1^{epi} \cdot R_2^{epi} \cdot (1 - X)}{X \cdot (R_1^{epi} - R_2^{epi})} + \Delta R^{epi} \cdot \frac{(R_1^{epi} + R_2^{epi} + \Delta R^{epi}) \cdot (1 - X)}{X \cdot (R_1^{epi} - R_2^{epi})} \qquad \text{(Eq. S8)}$$

This shows that ΔR^{epi} do not simply add up within this calculation, but have multiplicative impact. Consequently, calculation of R^{para} suffers considerably from even small deviations in R_1^{epi} and R_2^{epi}.

This effect can be quantified by its relative deviation. For determining the relative deviation, it is useful to define a deviation coefficient δ:

$$\delta = \frac{(R_1^{epi} + R_2^{epi} + \Delta R^{epi}) \cdot (1 - X)}{X \cdot (R_1^{epi} - R_2^{epi})} \qquad \text{(Eq. S9)}$$

Assuming again that ΔR_1^{epi} and ΔR_2^{epi} are of the same value, the relative deviation ΔR_{rel}^{para} is given by

$$\Delta R_{rel}^{para} = \frac{\Delta R^{epi} \cdot \delta}{R_1^{para}} = \Delta R^{epi} \cdot \left(\frac{R_1^{epi} + R_2^{epi} + \Delta R^{epi}}{R_1^{epi} \cdot R_2^{epi}} \right) = \Delta R^{epi} \cdot \frac{R_1^{epi} + R_2^{epi}}{R_1^{epi} \cdot R_2^{epi}} + \left(\Delta R^{epi}\right)^2 \cdot \frac{1}{R_1^{epi} \cdot R_2^{epi}} \qquad \text{(Eq. S10)}$$

As $\frac{R_1^{epi} + R_2^{epi}}{R_1^{epi} \cdot R_2^{epi}}$ is constant for given cells with given R_1^{epi} and R_2^{epi}, it can be concluded for the first term that:

$$\Delta R^{epi} \cdot \frac{R_1^{epi} + R_2^{epi}}{R_1^{epi} \cdot R_2^{epi}} \in \Omega\left(\Delta R^{epi}\right) \qquad \text{(Eq. S11)}$$

where Ω denotes Landau's symbol, meaning ΔR^{epi} as a function is a lower boundary for ΔR_{rel}^{para}.

Analogously, $\frac{1}{R_1^{epi} \cdot R_2^{epi}}$ is constant and it follows for the second term:

$$\left(\Delta R^{epi}\right)^2 \cdot \frac{1}{R_1^{epi} \cdot R_2^{epi}} \in \Omega\left(\left(\Delta R^{epi}\right)^2\right) \qquad \text{(Eq. S12)}$$

This implies that ΔR_{rel}^{para} grows at least at the order of the deviation of the estimated R^{epi} from the target value:

$$\Delta R_{rel}^{para} \in \Omega\left(\Delta R^{epi}\right) \qquad \text{(Eq. S13)}$$

Therefore, the overall growth of the relative error of R^{para} estimates can be considered at least linear and primarily dependend on ΔR^{epi}.

References

Krug, S. M., M. Fromm, and D. Günzel. 2009. Two-path impedance spectroscopy for measuring paracellular and transcellular epithelial resistance. *Biophys. J.* 97:2202–2211.
