# Supplementary material for: Discerning Apical and Basolateral Properties of HT-29/B6 and IPEC-J2 Cell Layers by Impedance Spectroscopy, Mathematical Modeling and Machine Learning
Source: PLoS One. 2013 Jul 1;8(7):e62913. doi: 10.1371/journal.pone.0062913 (PMC3698131; doi:10.1371/journal.pone.0062913)
Supplement: File S3 — Datasets overview: preprocessing, benchmarking data, frequencies. (PDF) [file pone.0062913.s015.pdf]

Datasets overview

Two different epithelial cell lines (HT-29/B6, IPEC-J2) were measured under two different settings (before EGTA application, after EGTA application). Therefore, four experimental conditions were consideredthat will be abbreviated as HT, HT+EGTA, IPEC, IPEC+EGTA.

For each condition, impedance spectra were measured and modeled (Tables S5 and S1, see also materials and methods section). Each measured sample consists of a total of 84 features resulting from a pairwise ordering of real and imaginary parts of impedances obtained at 42 different frequencies. Computationally created samples were modeled to match this characteristic. All datasets included in this work were complete (i.e. there were no missing values).

As target value for a given cell condition, either epithelial resistance or subepithelial resistance were considered; this results in a total of eight application scenarios and eight ANNs employed in the present study. The unit of the target domain in all cases is Ohm*cm^2.

All datasets are available upon request.

Preprocessing. As sigmoidal activation functions were employed in the hidden layer of the ANNs (cf. methods and materials section and Fig. S3), input and output of the ANNs were squashed by the used ANN simulator. Features of all training datasets were squashed between -1 and 1. Targets of all training datasets were squashed between the upper limit of the output activation function minus the sigmoidal offset and the lower limit plus the offset. These squashing settings were stored for each ANN individually, and applied to test data accordingly.

Benchmarking data. Measured datasets listed in Table S5 were used for benchmarking R^{sub} and R^{epi} estimations (plausibility of the electric model and the setup-specific error model). For evaluation with M1 and M2 all 84 values of each dataset were used, whereas for evaluation with ANNs, only the impedance values derived from the 10 highest (R^{sub}) and 10 lowest frequencies (R^{epi}) were used.

Similarly, 2x1000 benchmarking samples were derived from test samples of modeled datasets listed in Tables S2 and S3. These datasets were evaluated with M1 and M2 (all 84 values of each dataset) and with ANNs. For ANNs, only 20 values (R^{sub}, curve features 65-84 = real and imaginary parts of the impedance values calculated from the 10 highest frequencies; R^{epi}, curve features 1-20 = real and imaginary parts of the impedance values vcalculated from the 10 lowest frequencies) were used.

Frequencies. For impedance spectroscopy measurements and for the generation of model impedance spectra, n=42 frequencies $\omega = 2\cdot\pi\cdot f$ were employed. Lowest frequency f was 1.3 Hz, higher frequencies were multiples by a factor of $10^{0.1}$ (~1.26), resulting in 10 different frequencies per decade. The 10 lowest (f = 1.3 Hz to 10.326 Hz) and highest (f = 2.0603 kHz to 16.35 kHz) frequencies were used to create ANNs for R^{epi} and R^{sub}, respectively.
