# Supplementary material for: Discerning Apical and Basolateral Properties of HT-29/B6 and IPEC-J2 Cell Layers by Impedance Spectroscopy, Mathematical Modeling and Machine Learning
Source: PLoS One. 2013 Jul 1;8(7):e62913. doi: 10.1371/journal.pone.0062913 (PMC3698131; doi:10.1371/journal.pone.0062913)
Supplement: File S4 — Error model; Eq. S14–Eq. S17. (PDF) [file pone.0062913.s016.pdf]

Error model

Data scatter added to theoretical impedance spectra was modeled as function of corresponding frequency and transepithelial resistance R^T. The model is based on standard deviations (SDs) of Z^{re} and Z^{im}, respectively, which were modeled for each frequency and expressed as % of the DC resistance value.

For Z^{re} at frequency f, a second-order Fourier series (n=2) was employed:

$$SD_{re}(f) = a_0 + \sum_{i=1}^{n=2} a_i \cdot \cos(nwf) + b_i \cdot \sin(nwf) \qquad \text{(Eq. S14)}$$

where $w=5.353*10^{-5}$, $a_0=4.848$, $a_1=-4.11$, $b_1=-0.8092$, $a_2=-0.3583$, and $b_2=0.2014$ were determined as best fit to the measured data.

For Z^{im} at frequency f, a fourth-order polynomial function (n=4) was used:

$$SD_{im}(f) = a_0 + \sum_{i=1}^{n=4} a_i \cdot f^i \qquad \text{(Eq. S15)}$$

where $a_0=0.1889$, $a_1=0.0002737$, $a_2=1.863\cdot10^{-9}$, $a_3=-1.906\cdot10^{-13}$, $a_4=2.267\cdot10^{-18}$ were determined as best fit to the measured data.

To account for dependence of data scatter on R^T, SD_{re} and SD_{im} dynamics at 1.3 Hz were approximated by:

$$SD_{re}(1.3Hz) = 0.636^{R^T} - 0.3278 \qquad \text{(Eq. S16)}$$

$$SD_{im}(1.3Hz) = 8.7008^{R^T} - 0.8689 \qquad \text{(Eq. S17)}$$

This model was used to substitute a_0 in Eqs. A1 and A2 with $a_0(R^T) = a_0 + SD_{re}(1.3Hz)$ and $a_0(R^T) = a_0 + SD_{im}(1.3Hz)$, respectively, where a_0 (obtained from $R^T \approx 500\ \Omega\cdot cm^2$) had been normalized by $SD_{re}(1.3Hz)$ or $SD_{im}(1.3Hz)$ obtained at $R^T \approx 500\ \Omega\cdot cm^2$, respectively.
